# Determinants of unmet social needs and the role of parental mental health in families from multicultural and regional/rural communities of Australia

**DOI:** 10.1371/journal.pone.0332560

**Published:** 2026-08-11

**Authors:** James John, Teresa Winata, Si Wang, Melissa Smead, Weng Tong Wu, Jane Kohlhoff, Virginia Schmied, Bin Jalaludin, Kenny Lawson, Siaw-Teng Liaw, Raghu Lingam, Andrew Page, Christa Lam-Cassettari, Katherine Boydell, Ping-I. Lin, Ilan Katz, Ann Dadich, Shanti Raman, Rebekah Grace, Aunty Kerrie Doyle, Tom McClean, Blaise Di Mento, John Preddy, Susan Woolfenden, Valsamma Eapen

**Affiliations:** 1 School of Clinical Medicine, Faculty of Medicine and Health, University of New South Wales, Sydney, New South Wales, Australia; 2 Ingham Institute for Applied Medical Research, Liverpool, New South Wales, Australia; 3 Academic Unit of Infant, Child, and Adolescent Psychiatry Services, South Western Sydney Local Health District, Sydney, New South Wales, Australia; 4 National Disability Insurance Scheme Quality and Safeguards Commission, Parramatta, New South Wales, Australia; 5 Research and Evaluation Group, The Salvation Army, Sydney, New South Wales, Australia; 6 Murrumbidgee Local Health District, Wagga Wagga, New South Wales, Australia; 7 Karitane, Carramar, New South Wales, Australia; 8 School of Nursing and Midwifery, Western Sydney University, Sydney, New South Wales, Australia; 9 South Western Sydney Local Health District, Sydney, New South Wales, Australia; 10 School of Public Health and Community Medicine, Faculty of Medicine and Health, University of New South Wales, Kensington, New South Wales, Australia; 11 School of Medicine, Western Sydney University, Sydney, New South Wales, Australia; 12 WHO Collaborating Centre for eHealth, University of New South Wales, Kensington, New South Wales, Australia; 13 Population Child Health Research Group, School of Women’s and Children’s Health, Faculty of Medicine, University of New South Wales, Kensington, New South Wales, Australia; 14 Black Dog Institute, Sydney, Australia; 15 Department of Psychiatry and Behavioural Neuroscience, School of Medicine, Saint Louis University, Saint Louis, Missouri, United States of America; 16 Social Policy Research Centre, Faculty of Arts, Design, and Architecture, University of New South Wales, Kensington, New South Wales, Australia; 17 School of Business, Western Sydney University, Sydney, New South Wales, Australia; 18 Transforming early Education and Child Health Research Centre, Western Sydney University, Sydney, New South Wales, Australia; 19 Uniting, Sydney, New South Wales, Australia; 20 Rural Clinical School, School of Clinical Medicine, University of New South Wales, New South Wales, Australia; 21 Sydney Medical School, Faculty of Medicine and Health, University of Sydney, Sydney, New South Wales, Australia; 22 Sydney Local Health District, Sydney, New South Wales, Australia; Wollega University, ETHIOPIA

## Abstract

**Introduction:**

Families from disadvantaged communities often experience social care needs that adversely impact access to social and healthcare services. This study aimed to explore the determinants of social care needs and the associated clinical characteristics such as parental mental health among families from multicultural and regional/rural communities of Australia.

**Methods:**

This study is a secondary analysis of a randomised controlled trial conducted among parents/carers of children from culturally and linguistically diverse (CALD) communities of South Western Sydney and rural/regional communities of Murrumbidgee. The primary outcome of unmet social care needs was measured using the WE CARE survey. As the data were overdispersed (variance-to-mean ratio = 2.06), multivariable and generalised estimating equations (GEE) negative binomial regression models were applied to examine factors associated with unmet needs, with unmet needs treated as count outcomes.

**Results:**

Of the sample of 288 participants, 61% (n = 176) reported one or more unmet needs. Findings of the multivariable negative binomial regression analyses showed that clinical indicators such as parental mental distress (AIRR 1.05, 95% CI 1.04, 1.07) and child developmental concerns (AIRR 1.28, 95% CI 1.12, 1.45) alongside other sociodemographic factors such as CALD status (AIRR 1.63, 95% CI 1.13, 2.35), lower levels parental education (AIRR 2.25, 95% CI 1.62, 3.15), and marital status – De facto/single/divorced (AIRR 1.42, 95% CI 1.06, 1.89) were associated with higher rate of unmet needs at baseline. Additionally, findings of the GEE negative binomial model were largely consistent with the multivariable analyses and further demonstrated that families in the intervention group had a significantly lower rate of unmet needs over time compared with the control group (AIRR 0.75, 95% CI 0.60, 0.95).

**Conclusion:**

The study highlights the significant burden of unmet social needs among families from multicultural and rural/regional communities, emphasising the role of parental mental health and education levels as key contributing factors amongst other sociodemographic factors. Findings suggest the need for integrated, family-centred interventions that address both social and healthcare needs, particularly for vulnerable populations.

**Trial registration:**

This trial was registered with the Australian New Zealand Clinical Trials Registry (registration number: ACTRN12621000766819).

## Introduction

Unmet social care needs refer to the absence of critical resources such as housing, food security, employment, and access to mental and physical healthcare [1]. These challenges are exacerbated among families from priority population groups such as those from culturally and linguistically diverse (CALD) as well as regional or rural communities, where systemic barriers and socioeconomic disparities often limit access to social and healthcare services. In Australia, multicultural families, including migrants and refugees, face unique challenges due to language barriers, cultural adaptation, and discrimination, which can hinder their ability to access services [2,3]. Similarly, families in regional and rural areas encounter inequity in access due to geographic location, fewer healthcare facilities, and limited employment opportunities, exacerbating their social and economic vulnerabilities [4,5]. Hence, addressing these unmet needs is crucial, as they are strongly linked to poorer physical and mental health outcomes, intergenerational disadvantage, and reduced quality of life.

Australian health and social policies have emphasised the need for integrated, community-based approaches that address both medical and social determinants of health [6–9]. Despite these efforts, gaps persist in reaching high-risk populations, particularly those from the priority population groups. Existing service models often operate in silos, failing to provide comprehensive, coordinated care that addresses the multifaceted needs of families facing social disadvantage [10,11]. The social determinants of health (SDOH) framework provides a comprehensive approach for understanding how social, economic, and environmental factors collectively shape health outcomes. Studies have demonstrated the effectiveness of early intervention programs that address social determinants of health, leading to improved health and wellbeing and cost beneficial outcomes [12,13]. However, there is limited research on the specific factors and extent of unmet social needs and the interaction with parental mental health status and child developmental concerns among multicultural and regional/rural families in Australia.

To address this knowledge gap, this study aimed to determine the sociodemographic, sociocultural, and clinical indicators such as child developmental concerns and parental mental health associated with unmet social needs among families from multicultural and regional/rural communities of Australia. Findings of this study will provide critical insights into the barriers these families face in accessing essential social and healthcare supports. The evidence generated will inform targeted policy reforms and the development of interventions aimed at enhancing service integration and accessibility. Further, identifying and addressing these critical social care needs is crucial for promoting social equity, improving health outcomes, and ensuring that all Australian families have the support they need to thrive, regardless of their cultural, socioeconomic, and geographic background.

## Methods

### Human ethics and consent to participate declarations

The study conforms to the principles outlined in the Declaration of Helsinki. All methods were carried out in accordance with relevant guidelines and regulations of The National Statement on Ethical Conduct in Human Research (2023). The South Western Sydney Local Health District Human Research Ethics Committee approved this study (2020/ETH01418). All participating parents have provided written informed consent prior to participation. Further details are presented in the study protocol (S1 File).

### Study design and participants

Study findings are reported following the Consolidated Standards of Reporting Trials (CONSORT) guidelines (S2 File) [14]. This study is a secondary analysis of data from a parallel group randomised controlled trial (RCT) conducted among two priority population communities of South Western Sydney Local Health District (SWSLHD) and Murrumbidgee Local Health District (MLHD) representing predominantly multicultural/low-income and rural/regional communities, respectively. The authors confirm that all ongoing and related trials for this intervention are registered (ACTRN12621000766819). Parents/caregivers with a child aged six months to three years old attending Child and Family Health Services (CFHS) and other services providing child and family health care were recruited to participate in this study. All participants provided written informed consent. The recruitment and follow-up period of this study was between 1 August 2021 and 30 June 2023 with the recruitment commencing at the same time across both study sites. Further information on the randomisation and blinding, sample size calculation, and the intervention components are detailed in the published protocol [15].

### Outcome measure – Unmet social needs

Unmet social care needs in this study were assessed using the WE CARE survey, a brief and validated screening tool designed to efficiently identify the presence and extent of unmet social needs in families [16]. The survey comprises six binary (yes/no) responses related to family psychosocial needs, covering areas such as childcare, employment, homelessness, food security, education, and utilities. Additional questions were included for participants who respond affirmatively to any of the initial six items, prompting participants to indicate, on a three-point scale (yes, no, or maybe later), whether they require further assistance in addressing their unmet psychosocial needs. For the purpose of this study, negative binomial regression was then conducted to examine factors associated with unmet needs, treating them as count data given the overdispersion in the dataset.

### Explanatory variables

The clinical and health service use indicators included number of concerns reported via the Learn the Signs Act Early (LTSAE) assessment, parental mental health assessed via Kessler’s psychological distress scale (K10), and current service use (no, yes). The LTSAE is developed by the Centres for Disease Control and Prevention (CDC) [17], to monitor child’s development from 2 months to 5 years old. The assessment includes several domains of social and emotional, language/communication, cognitive, and movement/physical development. The K10 assessment is a widely used self-report questionnaire designed to measure psychological distress in individuals [18]. It comprises 10 items with ratings on a five-point scale assessing various aspects of emotional wellbeing and mental health over the past four weeks.

The sociodemographic and sociocultural factors included: child’s age (in months), child’s gender (female, male), parent’s age (in years), CALD status (yes, no), highest level of education (Postgraduate/graduate diploma/ Bachelors, Advance diploma/Certificate ¾, Year 12 and/or below), marital status (Married, others – de facto/single/divorced), IRSAD quintiles (from Q1 - most disadvantaged to Q5 - the most advantaged), and treatment group (intervention, control).

### Statistical analysis

Descriptive characteristics of the sample was computed using mean with standard deviation (SD) for continuous variables and frequency counts with percentages for categorical variables, with 95% confidence intervals estimated for all proportions. Shapiro-Wilks test for normality and analysis of normal quantile-quantile plots were used to assess the normality of distribution. Multivariable negative binomial regression analysis was used to determine the factors associated with increased rate of unmet needs, where unmet needs were treated as count data for each unmet need. Factors included sociodemographic, sociocultural, and clinical indicators where unmet social needs at baseline (model 1), 6 months (model 2), and 12 months (model 3) were the respective outcomes. Negative binomial regression was used due to overdispersion seen between the outcome and each of the above factors.

To assess the robustness of these findings and to account for repeated measurements over time, a Generalised Estimating Equations (GEE) negative binomial model with an exchangeable correlation structure was additionally fitted. This model incorporated all three time points (baseline, 6 months, and 12 months) and accounted for within‑participant clustering. Treatment group allocation (intervention vs control) was included as a covariate to reflect the underlying randomised controlled trial design.

Model diagnostics were conducted to assess the suitability of the count regression models. Overdispersion was evaluated using the variance-to-mean ratio, multicollinearity was assessed using Variance Inflation Factors (VIFs), and model fit was examined through inspection of Pearson residuals and fitted-value plots.

All analyses were undertaken in Stata v19 (StataCorp. 2025. *Stata Statistical Software: Release 19*. College Station, TX: StataCorp LLC.)

## Results

### Participant characteristics

**Fig 1:**
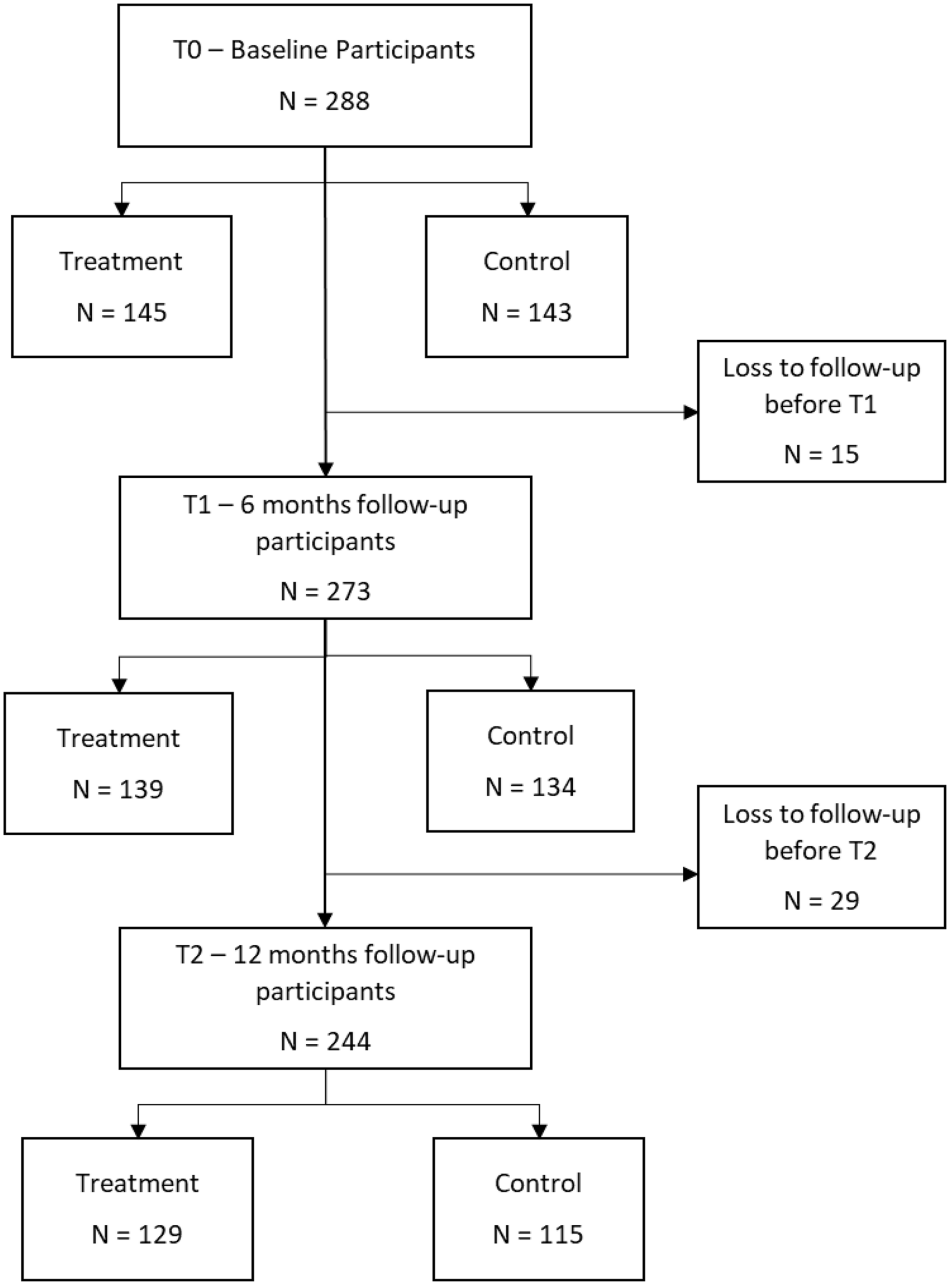
CONSORT Study Flow Diagram.

The CONSORT flowchart showing randomisation of participants along with follow-up at different time points is summarised in Fig 1. The descriptive characteristics of the sample at baseline and the prevalence of the unmet needs status by background characteristics are presented in Tables 1 and 2. Of the total sample of 288 participants, 61% (n = 176) reported one or more unmet needs at baseline with the top concerns being help with food, mental health, employment, and day care.

**Table 1:** Descriptive characteristics of participants at baseline.

Variables	Total sample (N = 288) n (%) or M(SD)
**Sociodemographic and sociocultural factors**	
Child’s age in months	
*Infants (up to 11 months)*	83 (28.8)
*Toddlers (12–36 months)*	205 (71.2)
Child’s gender	
*Female*	145 (50.3)
*Male*	143 (49.7)
Parent/Caregiver’s age in years	31.23 (6.50)
*<25 years*	36 (12.5)
*≥25 years*	252 (87.5)
CALD status^#^	
*No*	206 (71.5)
*Yes*	46 (16.0)
*Missing*	36 (12.5)
Parent/Caregiver’s education level	
*Postgraduate/Graduate diploma/Bachelor’s degree*	148 (51.4)
*Advanced Diploma/ Certificate III/IV*	78 (27.1)
*Up to Year 12*	62 (21.5)
Marital status	
*Married*	178 (61.8)
*De facto/Single/Divorced*	109 (37.8)
*Missing*	1 (0.4)
Socioeconomic status (IRSAD)	
*Quintiles 1–2 (disadvantaged)*	160 (55.6)
*Quintile 3*	109 (37.8)
*Quintiles 4–5 (advantaged)*	19 (6.6)
Treatment group	
*Control*	143 (49.7)
*Intervention*	145 (50.3)
**Clinical indicators and health service use**	
No of child developmental concerns	0.27 (0.66)
*No concerns*	235 (81.6)
*One or more concerns*	53 (18.4)
Parental mental wellbeing (K10 scores)	18.47 (6.78)
*Likely to be well*	184 (63.9)
*Mild mental disorder*	49 (17.0)
*Moderate mental disorder*	29 (10.1)
*Severe mental disorder*	26 (9.0)
Premature birth	
*No*	272 (94.4)
*Yes*	16 (5.6)
Current service use	
*No*	211 (73.3)
*Yes*	73 (25.3)
*Missing*	4 (1.4)

^#^CALD status – parent’s country of birth and/or preferred language; n – number/count; % - proportion; M – mean; SD – standard deviation.

**Table 2:** Prevalence of one or more unmet needs by background characteristics.

Variables	No unmet needs (N = 112) n (%) or M(SD)	95% CI	One or more unmet needs (N = 176) n (%) or M(SD)	95% CI
**Sociodemographic and sociocultural factors**				
Child’s age in months				
*Infants (up to 11 months)*	34 (41.0)	30.4-51.5	49 (59.0)	48.5-69.6
*Toddlers (12–36 months)*	78 (38.0)	31.4-44.5	127 (62.0)	55.3-68.6
Child’s gender				
*Female*	58 (40.0)	32.0-47.9	87 (60.0)	52.0-67.9
*Male*	54 (38.0)	29.8-45.7	89 (62.0)	54.3-70.2
Parent/Caregiver’s age in years				
*<25 years*	6 (16.7)	4.5-28.8	30 (83.3)	71.2-95.5
*≥25 years*	106 (42.1)	35.9-48.2	146 (57.9)	51.8-64.0
CALD status^#^				
*No*	87 (42.0)	35.5-48.9	119 (58.0)	51.0-64.5
*Yes*	16 (35.0)	21.0-48.5	30 (65.0)	51.5-78.9
*Missing*	9 (25.0)	10.9-39.2	27 (75.0)	60.9-89.2
Parent/Caregiver’s education level				
*Postgraduate/Graduate diploma/Bachelor’s degree*	71 (48.0)	39.9-56.0	77 (52.0)	43.9-60.1
*Advanced Diploma/ Certificate III/IV*	33 (42.0)	31.3-53.3	45 (58.0)	46.7-68.7
*Up to Year 12*	8 (13.0)	4.6-21.3	54 (87.0)	78.8-95.4
Marital status				
*Married*	85 (48.0)	40.4-55.1	93 (52.0)	44.9-59.6
*De facto/Single/Divorced*	27 (25.0)	16.7-32.9	82 (75.0)	67.1-83.3
*Missing*	0 (0.0)	0.0-0.0	1 (100.0)	100.0-100.0
Socioeconomic status (IRSAD)				
*Quintiles 1–2 (disadvantaged)*	65 (41.0)	33.0-48.2	95 (59.0)	51.8-66.9
*Quintile 3*	37 (34.0)	25.1-42.8	72 (66.0)	57.2-74.9
*Quintiles 4–5 (advantaged)*	10 (53.0)	30.2-75.1	9 (47.0)	24.9-69.8
Treatment group				
*Control*	45 (31.0)	23.9-39.1	98 (69.0)	60.9-76.1
*Intervention*	67 (46.0)	38.1-54.3	78 (54.0)	45.7-61.9
**Clinical indicators and health service use**				
No of child developmental concerns				
*No concerns*	100 (42.6)	36.2-48.9	135 (57.4)	51.1-63.8
*One or more concerns*	12 (22.6)	11.4-33.9	41 (77.4)	66.1-88.6
Parental mental wellbeing (K10 scores)				
*Likely to be well*	87 (47.3)	40.1–54.5	97 (52.7)	45.5–59.9
*Mild mental disorder*	19 (38.8)	25.1–52.4	30 (61.2)	47.6–74.8
*Moderate mental disorder*	4 (13.8)	1.2–26.3	25 (86.2)	73.7–98.7
*Severe mental disorder*	2 (7.7)	0.0–17.9	24 (92.3)	82.1–100.0
Premature birth				
*No*	107 (39.0)	33.5-45.1	165 (61.0)	54.9-66.5
*Yes*	5 (31.0)	8.5-53.9	11 (69.0)	46.0-91.5
Current service use				
*No*	80 (38.0)	31.4-44.5	131 (62.0)	55.5-68.6
*Yes*	30 (41.0)	29.8-52.4	43 (59.0)	47.6-70.2
*Missing*	2 (50.0)	1.0-99.0	2 (50.0)	1.0-99.0

### Findings of the regression analyses

Findings of the multivariable negative binomial regression models (Table 3) showed that higher scores on parental mental health issues via K10 (AIRR 1.28, 95% CI: 1.12, 1.45) and higher number of child developmental concerns (AIRR 1.05, 95% CI: 1.04, 1.07) were associated with higher rate of unmet needs at baseline. In terms of sociodemographic and sociocultural factors, those from a CALD background had 63% higher rate of unmet needs at baseline (AIRR 1.63, 95% CI: 1.13, 2.35). Lower level of parental education (up to year 12) were associated with twice as higher rate of unmet needs (AIRR 2.25, 95% CI: 1.62, 3.15) at baseline compared to those with tertiary level education. Compared to parents/caregivers who were married, those who were de facto, single, or divorced had 42% higher rate of unmet needs (AIRR 1.42, 95% CI: 1.06, 1.89) at baseline. Finally, consistent with the univariable analysis, older parents (parents who were equal to or over 25 years) were associated with lower rate of unmet needs (AIRR 0.80, 95% CI: 0.56, 1.13) at baseline.

**Table 3:** Associations between sociodemographic factors, clinical factors, and unmet social needs (adjusted models) (Multivariable negative binomial regression models).

Variables	At baseline AIRR (95% CI)	At 6 months AIRR (95% CI)	At 12 months AIRR (95% CI)
Child’s age in months			
*Infants (up to 11 months)*	Reference category	Reference category	Reference category
*Toddlers (12–36 months)*	0.96 (0.61, 1.48)	1.40 (0.91, 2.34)	1.40 (0.81, 2.39)
Child’s gender			
*Female*	Reference category	Reference category	Reference category
*Male*	0.99 (0.77, 1.27)	1.12 (0.81, 1.55)	1.13 (0.78, 1.64)
Parent/Caregiver’s age in years			
*<25 years*	Reference category	Reference category	Reference category
*≥25 years*	0.80 (0.56,1.13)*	0.60 (0.36, 0.99)*	0.75 (0.43, 1.31)
CALD status^#^			
*No*	Reference category	Reference category	Reference category
*Yes*	1.63 (1.13, 2.35)*	1.55 (0.99, 2.45)	1.46 (0.86, 2.51)
Parent/Caregiver’s education level			
*Postgraduate/Graduate diploma/Bachelor’s degree*	Reference category	Reference category	Reference category
*Advanced Diploma/ Certificate III/IV*	1.08 (0.76, 1.54)	1.36 (0.89, 2.07)	1.57 (0.98, 2.53)
*Up to Year 12*	2.25 (1.62, 3.15)*	2.28 (1.47, 3.51)*	1.98 (1.19, 3.31)*
Marital status			
*Married*	Reference category	Reference category	Reference category
*De facto/Single/Divorced*	1.42 (1.06, 1.89)*	1.44 (1.01, 2.08)*	1.44 (0.94, 2.20)
Socioeconomic status (IRSAD)			
*Quintiles 1–2 (disadvantaged)*	Reference category	Reference category	Reference category
*Quintile 3*	1.01 (0.77, 1.33)	1.11 (0.77, 1.58)	0.94 (0.63, 1.40)
*Quintiles 4–5 (advantaged)*	0.71 (0.36, 1.40)	1.11 (0.54, 2.27)	0.67 (0.24, 1.84)
Treatment group			
*Control*	Reference category	Reference category	Reference category
*Intervention*	0.80 (0.63, 1.03)	0.84 (0.61, 1.17)	0.79 (0.54, 1.15)
**Clinical indicators and health service use at baseline**			
No of child developmental concerns, mean (SD)	1.28 (1.12, 1.45)*	1.13 (0.92, 1.39)	1.13 (0.90, 1.41)
Parental mental wellbeing (K10 scores), mean (SD)	1.05 (1.04, 1.07)*	1.03 (1.01, 1.06)*	1.05 (1.03, 1.08)*
Premature birth			
*No*	Reference category	Reference category	Reference category
*Yes*	1.10 (0.64, 1.91)	0.90 (0.44, 1.87)	1.60 (0.81, 3.20)
Current service use			
*No*	Reference category	Reference category	Reference category
*Yes*	1.01 (0.76, 1.34)	1.05 (0.74, 1.49)	1.43 (0.97, 2.10)

AIRR – Adjusted Incidence Rate Ratio from Multivariable Negative Binomial Regression; CI – Confidence Intervals; *p-value<0.05.

Similar to model 1 (baseline), higher parental mental health distress and lower levels of parental education were significantly associated with higher rate of unmet needs at 6 and 12 months (models 2 and 3) whereas marital status (de facto, single, or divorced) and younger parents were significantly associated with higher rate of unmet needs at 6 months (model 2). Current service use did not moderate the association between CALD, parental education, marital status, and unmet needs.

To assess the robustness of these findings and account for repeated measurements over time, a GEE negative binomial model was fitted (Table 4). The GEE model incorporated all three time points simultaneously and adjusted for within-participant clustering. The results were highly consistent with the standalone negative binomial models. Significant predictors in the GEE model included parental psychological distress, number of child developmental concerns, CALD background, lower parental education, marital status, and parental age. The intervention group showed a significantly lower rate of unmet needs (AIRR 0.75, 95% CI: 0.60, 0.95), consistent with the direction of effects observed in the original models.

**Table 4:** Associations Between Sociodemographic and Clinical Factors and Unmet Social Needs (GEE Negative Binomial Model).

Variables	AIRR (95% CI)
Child’s age in months	
*Infants (up to 11 months)*	Reference category
*Toddlers (12–36 months)*	1.23 (0.94, 1.62)
Child’s gender	
*Female*	Reference category
*Male*	0.99 (0.78, 1.25)
Parent/Caregiver’s age in years	
*<25 years*	Reference category
*≥25 years*	0.65 (0.43, 0.97) *
CALD status^#^	
*No*	Reference category
*Yes*	1.57 (1.11, 2.20) *
Parent/Caregiver’s education level	
*Postgraduate/Graduate diploma/Bachelor’s degree*	Reference category
*Advanced Diploma/ Certificate III/IV*	1.27 (0.94, 1.72)
*Up to Year 12*	2.40 (1.76, 3.28) *
Marital status	
*Married*	Reference category
*De facto/Single/Divorced*	1.45 (1.11, 1.9) *
Socioeconomic status (IRSAD)	
*Quintiles 1–2 (disadvantaged)*	Reference category
*Quintile 3*	1.17 (0.89, 1.54)
*Quintiles 4–5 (advantaged)*	0.97 (0.59, 1.60)
Treatment group	
*Control*	Reference category
*Intervention*	0.75 (0.60, 0.95) *
**Clinical indicators and health service use at baseline**	
No of child developmental concerns, mean (SD)	1.33 (1.14, 1.55) *
Parental mental wellbeing (K10 scores), mean (SD)	1.03 (1.01, 1.05) *
Premature birth	
*No*	Reference category
*Yes*	1.18 (0.73, 1.93)
Current service use	
*No*	Reference category
*Yes*	1.09 (0.83, 1.43)

AIRR – Adjusted Incidence Rate Ratio from GEE Negative Binomial Regression; CI – Confidence Intervals; *p-value<0.05; All effects are controlled for the time factor (baseline, 6-months, and 12-months).

**Fig 2:**
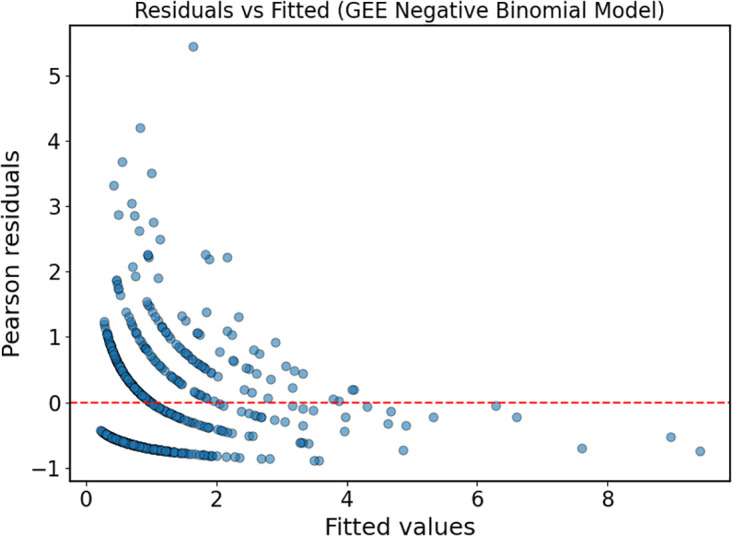
Diagnostic plots, including Pearson residual distributions and residual‑versus‑fitted values.

**Fig 3:**
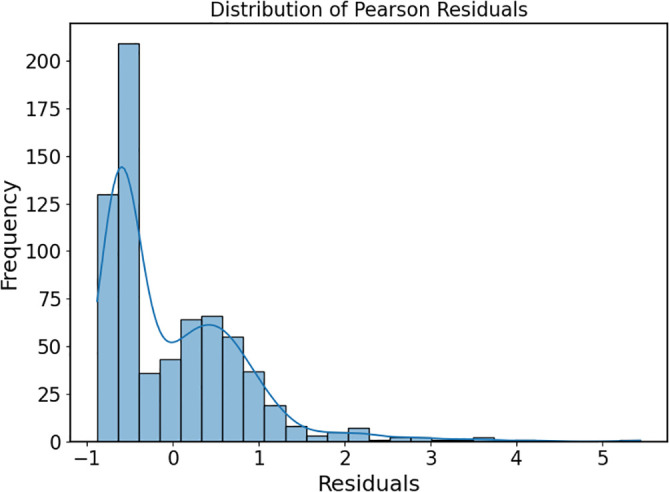
Distribution of Pearson residuals.

Diagnostic evaluation supported the adequacy of both modelling approaches (Figs 2 and 3). Evidence of overdispersion was observed (variance-to-mean ratio = 2.06), supporting the use of count regression models. Multicollinearity was low, with all Variance Inflation Factors (VIFs) below 2. Inspection of Pearson residuals and fitted-value plots indicated acceptable model fit and no major violations of model assumptions.

## Discussion

This study aimed to identify the sociodemographic, sociocultural, and clinical indicators of unmet social needs among families from CALD and regional/rural communities in Australia. Our findings highlighted that a substantial proportion (61%) of families reported at least one unmet social need at baseline, with food insecurity, employment, and daycare needs being the most prevalent concerns. Additionally, parental mental health issues and child developmental concerns alongside key social and clinical risk factors such as CALD status, parental education, and marital status were found to be significantly associated with unmet needs. Additionally, findings of the GEE model further suggest that these factors may have a sustained influence on families’ unmet social needs over time, rather than reflecting challenges at baseline. This underscores the urgent need for targeted, culturally sensitive, and multifaceted interventions and policies to effectively address these complex social challenges and improve the wellbeing of vulnerable families across Australia.

Poor parental mental health was a critical determinant and was associated with increased rate of unmet needs. There is substantial evidence highlighting the bidirectional relationship where poor mental health can both contribute to and result from social disadvantage [19,20]. Parents experiencing psychological distress, including anxiety and depression, often face difficulties in fulfilling their caregiving roles effectively. Poor mental health can compromise their ability to meet their children’s basic needs, maintain stable housing, or navigate social and healthcare systems, thereby compounding existing social vulnerabilities [20–22]. Consequently, examining the role of parental mental health in predicting social disparities is vital for designing effective interventions. Understanding how poor mental health drives unmet social needs can inform the development of holistic, wraparound interventions that addresses both psychological wellbeing and social determinants concurrently, thereby reducing disparities and improving family outcomes over the long term.

We found that higher number of child developmental concerns were significantly associated with increased rate of unmet needs. Parents and carers may face considerable psychosocial and financial strain when supporting a child with developmental vulnerabilities, especially if the child has severe developmental needs, which can substantially affect the family’s overall wellbeing and resources [23]. In addition, families may encounter practical barriers such as long waiting times for services, difficulties navigating complex or fragmented support systems, lack of culturally appropriate resources, and limited awareness of available assistance which may contribute to the higher rate of unmet social care needs [24,25].

Consistent with previous research, lower parental education emerged as one of the strongest predictors of unmet social needs, with those having only up to year 12 education having twice as higher rate of unmet needs compared to parents with tertiary education. This highlights the critical role of education in providing families with health literacy, problem-solving, and access to services that can mitigate social vulnerabilities [26]. Further, it is well established that higher education is strongly linked to better employment opportunities and income stability, further reducing the risk of social disadvantage [27]. Consequently, policies and interventions aimed at reducing unmet social needs must prioritise addressing educational inequalities such as providing access and support to health literacy and community learning programs. By indirectly strengthening families’ resources and capacities through education, these initiatives have the potential to significantly alleviate social vulnerabilities and improve overall family outcomes [28].

We also found key sociocultural factors such as family structure, CALD status, and parental age to further influence the extent to which families experience unmet social needs. Marital status was a significant factor, where parents who were single, divorced, or in de facto relationships had higher rate of experiencing unmet needs compared to parents who were married. This finding aligns with literature suggesting that single-parent and non-traditional family structures often face greater financial and social pressures, which may limit their ability to meet essential needs [29–31]. Additionally, we found that older parents were likely to have lower rate of unmet needs compared to younger parents. This is consistent with previous findings [32] that older parents may have greater life experience, financial stability, and established social support networks compared to younger parents, which could enable them to more effectively navigate services and access resources, thereby reducing the likelihood of unmet needs. Notably, families from CALD backgrounds experienced higher rate of unmet needs, reiterating the substantial barriers faced by those from CALD communities. These barriers may include language difficulties, lack of familiarity with available services, and potential discrimination, restricting access to essential supports [2,33,34]. These findings highlight the need for culturally sensitive, family-centred policies and support systems that address the specific challenges faced by diverse family structures and CALD communities to reduce social inequities and improve access to critical resources.

Findings of the GEE analysis also demonstrated that families in the intervention group had lower rate of unmet needs over time, after accounting for repeated measurements over time and controlling for relevant sociodemographic and clinical factors. This finding suggests that systematic screening for social needs combined with tailored service navigation may be effective in helping families access resources and services that address their unmet needs. These findings are consistent with growing evidence that integrated, family-centred approaches addressing both social determinants and service accessibility can improve outcomes for vulnerable families and highlight the potential value of embedding social needs screening and navigation support within routine child and family health services [35,36].

### Strengths, limitations, directions for future research

This study has several strengths and limitations. One of the key strengths of this research is that it addresses a gap in understanding the determinants of unmet social needs in multicultural and regional/rural Australian families, populations often underrepresented in research. The use of validated measures for mental health and unmet needs enhances the reliability of findings. However, several limitations should be acknowledged. The reliance on self-reported data may introduce reporting bias or social desirability effects. Our sample, although diverse, may not fully represent all multicultural or regional/rural groups across Australia, limiting generalisability.

Future studies should aim to explore specific cultural, linguistic, and geographic barriers via qualitative research in greater detail to inform more nuanced and effective interventions for multicultural and regional/rural communities. Evaluations of digital and community-based navigation models over longer periods will help clarify optimal implementation strategies and sustainability. Finally, investigating policy-level interventions addressing structural determinants, such as housing and employment policies, will be crucial to comprehensively reducing unmet social needs.

### Implications for policy and practice

Our findings highlight the importance of tailored approaches to identify and address unmet social needs among vulnerable families. Policies should prioritise education as a key social determinant, integrating education and family support programs to empower parents. Mental health services should be embedded within community and social care frameworks, with culturally sensitive approaches and virtual support to meet the specific needs of CALD and rural/regional populations, respectively. Family support services must recognise individual family dynamics such as the additional challenges faced by single-parent and non-traditional families, ensuring access to flexible and affordable childcare, employment support, and food assistance. The demonstrated potential of digital screening and navigation interventions suggests that scaling such models could improve service access and reduce unmet needs, particularly when adapted for cultural and regional contexts.

## Conclusion

Our study provides valuable insights into the key factors associated with increased rate of unmet social needs among families from diverse CALD and regional/rural communities in Australia that often leads to inequity in access to services. The findings emphasise the complex and interconnected nature of sociodemographic, sociocultural, clinical factors that contribute to social vulnerabilities within these priority populations. It is imperative that policies and programs adopt a holistic, culturally sensitive approach that addresses social disparities and integrates mental health supports to overcome barriers to service access, in particular for culturally and linguistically diverse families. Further research and sustained investment in tailored interventions are critical to promoting equity and wellbeing for all Australian families.

## Supporting information

S1 File Detailed Study Protocol. (DOCX)

S2 File CONSORT checklist. (DOCX)
