# Supplementary material for: Determinants of unmet social needs and the role of parental mental health in families from multicultural and regional/rural communities of Australia
Source: PLoS One. 2026 Aug 11;21(8):e0332560. doi: 10.1371/journal.pone.0332560 (PMC13460624; doi:10.1371/journal.pone.0332560)
Supplement: Supplementary file 1 [file pone.0332560.s001.docx]

**PROTOCOL TITLE**

**A ‘Digital’ Support for Child and Family Health Services for developmental surveillance: Using the Watch Me Grow web-app to address a critical COVID-19 service delivery gap**

Protocol No. 1.0 (Version 4.2)

**Sponsor**

NSW Ministry of Health COVID-19 Research Grants Round 2

**TABLE OF CONTENTS**

[PROTOCOL TITLE	1](#_Toc66869491)

[Sponsor	1](#_Toc66869492)

[1. SUMMARY	8](#_Toc66869493)

[2. BACKGROUND AND RATIONALE	9](#_Toc66869494)

[3. STUDY AIMS/OBJECTIVES	10](#_Toc66869495)

[4. PARTICIPATING SITES	10](#_Toc66869496)

[5. STUDY DESIGN	10](#_Toc66869497)

[qualitative implementation evaluation	11](#_Toc66869498)

[5.1 Study Type	11](#_Toc66869499)

[5.2 Expected Study Duration	11](#_Toc66869500)

[5.3 Data Source and Population	12](#_Toc66869501)

[5.4 Recruitment and Screening	13](#_Toc66869502)

[5.5 Inclusion Criteria	14](#_Toc66869503)

[5.6 Exclusion Criteria	14](#_Toc66869504)

[5.7 Consent Process	14](#_Toc66869505)

[5.8 Study Procedures	15](#_Toc66869506)

[5.9	Randomisation	18](#_Toc66869507)

[5.10 Data Linkage (if applicable)	18](#_Toc66869508)

[6.	TISSUE CoLLECTION/BIOBANKING (IF APPLICABLE)	18](#_Toc66869509)

[7. ETHICAL CONSIDERATIONS	18](#_Toc66869510)

[7.1 Study Procedure Benefits	18](#_Toc66869511)

[7.2 Study Procedure Risks	19](#_Toc66869512)

[7.3 Confidentiality and Privacy	20](#_Toc66869513)

[7.4 Data Storage and Record Retention	20](#_Toc66869514)

[8.	SAFETY REPORTING (IF APPLICABLE)	20](#_Toc66869515)

[9.	Data Safety and Monitoring Board (Clinical Trials only)	21](#_Toc66869516)

[10.	Early Termination (IF APPLICABLE)	21](#_Toc66869517)

[11.	BLINDING AND UNBLINDING (if applicable)	21](#_Toc66869518)

[12. CONFLICT OF INTEREST	21](#_Toc66869519)

[13. FUNDING	21](#_Toc66869520)

[14. RESEARCH OUTCOMES	21](#_Toc66869521)

[15. REFERENCES	21](#_Toc66869522)

**1. SUMMARY**

| **Study Title** | A ‘Digital’ Support for Child and Family Health Services for developmental surveillance: Using the Watch Me Grow web-app to address a critical COVID-19 service delivery gap |
| --- | --- |
|  |  |
| **Aims/Objectives** | The NSW State Blue Book/Personal Health Record (PHR) developmental surveillance program is one of the critical preventative health programs in the preschool period but it has come to a standstill due to Child and Family Health Nurse (CFHN) clinics being mostly closed due to the COVID-19 pandemic. As a direct consequence of this, children are missing out on developmental checks, one of the critical preventative health programs in the preschool period. This ‘digital support’ for CFHS aims to compare a “Watch Me Grow – Electronic platform” (WMG-E), a web-app for digital developmental monitoring as an alternative to CFHN clinics, to determine whether it improves uptake of and ongoing engagement with the developmental surveillance program. The objectives of the project include:  1. Effectiveness: Does WMG-E identify and address parental mental health, psychosocial needs, and child development needs for families of children aged 0-5 from CALD and regional/rural communities?  2. Acceptability: evaluate/understand the experience of using WMG-E  3. Is WMG-E cost-effective? |
| **Study design** | 1. This study will use a randomised controlled trial design, with individuals randomized electronically to either care as usual (CaU; provision of resources) (control group) or WMG-E + CaU (connection to services). 2. A qualitative implementation evaluation involving interviews for parents/caregivers, CFHNs and other stakeholders. |
| **Planned sample size** | Assuming power=80%, alpha=0.05, and a medium effect size *(d =* 0.2) for within-between interactions in a repeated measures MANOVA with 2 groups and 3 measures the sample size should be 244. Taking non- and partial-adherence and dropout into account the require sample size was revised to 300 (150 per group)*.* |
| **Inclusion criteria** | Parent/caregiver with children aged 6 months to 3 years living in either the Murrumbidgee LHD or Fairfield, Liverpool and Bankstown LGAs attending CFHS, refugee health services, supported playgroups, parenting groups, Karitane services, GP practices, special paediatric clinics, or Sustaining NSW families programs |
| **Study procedures** | Participants will be randomised upon completion of the demographics form to either CaU or WMG-E + CaU. All participants will be screened for: (a) Mental health concerns, (b) Unmet psychosocial needs, or (c) Developmental concerns. Participants in both groups, depending on their, responses they will receive targeted resources, whereas participants in the WMG-E + CaU arm will also be linked to services and further assessments as needed by health professionals.  Families will be followed-up at 6 and 12-months using the same screening tools to study the long-term effectiveness of the interventions.  We will also conduct a qualitative implementation evaluation to measure the experience of using WMG-E using the Consolidated Framework for Implementation Research (CFIR) five implementation domains evaluating the acceptability, adoption, appropriateness, fidelity (use), coverage and sustainability of this equity focussed pathway by CFHNs. These outcomes will be explored via in-depth, richly textured qualitative semi-structured interviews. |
| **Analysis considerations** | The analysis will compare the three main measures across three timepoints (Baseline, 6-months, 12-months) and between the two groups (CaU vs. WMG-E+CaU) using a MANOVA. Secondary analyses include qualitative interviews (described above) and an economic evaluation, which will use generalised estimating equations (GEE) and ‘within-trial’ analyses will capture cost-effectiveness converting K-10 scores to health utilities using a mapping algorithm; and estimating affordability if the intervention is implemented routinely. |
| **Research outcomes** | Families in the intervention group are expected to show significantly improved parental mental health, psychosocial wellbeing, and child development outcomes as compared to CAU group at 6 and 12 months. By targeting CALD and rural communities, we will demonstrate its feasibility for the most vulnerable Australians that will then be ready for scaling up. WMG-E is also expected to demonstrate that leveraging off child health services successfully links families with mental health care and psychosocial support services. |
| **Study duration** | The project will run for 24 months, from April 2021 to April 2023. |

**2. BACKGROUND AND RATIONALE**

Early child development (including speech and language, motor, and cognitive development) is an important predictor of health, mental wellbeing, and school attainment. The increasing prevalence of developmental disorders in early childhood poses a significant global health burden [1,2] with an estimated 200 million children worldwide not reaching their developmental potential [3,4]. Data from the Australian Early Development Censuses (AEDC) indicate that around one in five children starting their first year of school are “developmentally vulnerable”, but the detection is often delayed. In this regard, a previous NHMRC funded Watch Me Grow (WMG) study [5-8] found that the period from 12 months to 5 years of age was a critical “silent” period for assessing developmental issues including speech and language problems and Autism Spectrum Disorder (ASD), resulting in missed opportunities for early intervention using the critical window of brain plasticity in the preschool years. Early intervention in the first few years of life is the most promising avenue to improve child development and mental health and lower family stress and dysfunction. The last three decades have seen significant data suggesting that programs beginning in infancy and toddler years have the potential to affect key developmental outcomes [8, 9,10] and, the earlier the intervention, the better the outcome.

The NSW State Blue Book/Personal Health Record (PHR) developmental surveillance program is *one of the critical preventative health programs* in the preschool period but it has come to a standstill due to Child and Family Health Nurse (CFHN) clinics being mostly closed due to the COVID-19 pandemic. Child development is time sensitive, and without early identification, risk can snowball into intractable neurodevelopmental conditions with lifelong consequences; estimated to cost the economy 27 billion/year. As a direct consequence of the significant reduction in CFHN clinics that are in operation, children are missing out on developmental checks, one of the critical preventative health programs in the preschool period.

This project will bed down a new and innovative solution to the current service delivery gap in developmental surveillance, ensuring that no child is left behind during this COVID-19 crisis (and beyond). This project will implement a scalable ‘**Digital Support’** for CFHS embedded within the existing health services *to identify/assess/monitor children at developmental risk, with unmet psychosocial risks, or whose parents are experiencing distress and recommend care pathways*. The ‘Digital Support’ will be delivered via a web-tool as an add-on to CFHN in-person clinics, thereby engaging parents at their homes to complete developmental screening measures (including translations) with an in-built algorithm to identify children deemed at-risk, with results transmitted to the relevant teams.

**3. STUDY AIMS/OBJECTIVES**

The aim of our research is to evaluate using a randomised controlled trial (RCT), 1) the acceptability, 2) effectiveness and 3) cost-effectiveness of the WMG-E compared to “care as usual” (CAU), to identify and address parental mental health, psychosocial needs and child developmental needs, for families of children aged 0-5 years from CALD and regional/rural communities.

**4. PARTICIPATING SITES**

The two participating sites are Murrumbidgee LHD and Fairfield, Liverpool and Bankstown LGAs. Within each of these sites CFHS, refugee health services, supported playgroups, parenting groups, Karitane services, GP practices, special paediatric clinics, or Sustaining NSW families programs.

**5. STUDY DESIGN**

**Randomised control trial (RCT)**

The study design is a two (group: WMG-E + CaU vs CaU) x 3 (time: baseline, post-assessment, and follow-up) RCT. The trial will be registered with ANZCTR and will adhere to CONSORT guidelines. All families in contact with services (i.e., CFHS, refugee health services, supported playgroups, parenting groups, Karitane services, GP practices, special paediatric clinics, or Sustaining NSW families programs) in Fairfield, Liverpool and Bankstown LGAs and Murrumbidgee LHD will receive a link to inform them about the study and participating parents will complete baseline measures using the link. Children and families will then be randomised (via web link) to either WMG-E + usual care (intervention group) or usual care only (CAU; control group). All participating families will be assessed at trial entry (baseline), post-assessment (6-months) and follow-up (12-months).

**Qualitative implementation evaluation**

The qualitative implementation evaluation involves interviews with parents/caregivers of infants/toddlers/preschool age children, CFHNs, allied health and other key stakeholders.

**5.1 Study Type**

This is a RCT involving approximately 300 participants with a qualitative implementation evaluation.

**5.2 Expected Study Duration**

The study will take 24 months (April 2021 – April 2023).

| **Key milestone** | **Related deliverables** | **Completion date (mm/yyyy)** |
| --- | --- | --- |
| Commence training CFHN units in the use of WMG-E | - Modify WMG-E to include: - Parental mental health screening - Psychosocial need screening - Training CFHNs in use of WMG-E web app   Commence recruitment to the study | 05/2021  (Month 1) |
| Recruitment of study staff and ethics amendment | - Recruitment and onboarding of study staff at each site   Submission of ethics amendment | 05/2021 – 06/2021  (Months 1-2) |
| Recruitment to the study complete | Recruitment of 300 parents of children aged 12-24 months old completed; baseline WMG-E measure complete | 07/2021 – 01/2022  (Month 3-9) |
| Follow-up with families commences for Time 2  (6-month timepoint) | - Families are presented with the WMG-E web app for follow-up completion of measures | 01/2022 – 07/2022  (Month 9-15) |
| Uptake assessed | Analyse date on uptake of training | 01/2022 – 02/2022  (Month 9-10) |
| Invitations to CFHN to participate in training and education | Recruitment of CFHNs to participate in training and education activities to effectively scale the research | 02/2022 – 04/2022  (Month 10-12)  **Trial midpoint** |
| Follow-up with families commences for Time 3 (12-month timepoint);  Continue to carry out training with CFHN | - Families are presented with the WMG-E web app for follow-up completion of measures - Training conducted with CFHNs | 07/2022 – 01/2023  (Month 14-20) |
| Knowledge translation activities commence | Preliminary and acceptability findings translated into different art pieces, etc. to ensure community engagement | 06/2022 – 08/2022  (Month 13-15) |
| Preliminary analysis of findings | - Analyse preliminary findings to assess whether the families coming in are experiencing the expected improvements - Ensure follow-up assessments are being carried out smoothly | 08/2022 – 02/2023  (Month 15-21) |
| Implementation 1: Ensure compatibility with NSW Health systems | - Ensure tools used are compatible with NSW Health protocols and procedures, paving the way towards implementation | 11/2022  (Month 18) |
| Final follow up with families; analyse data and prepare final findings | - Final completion of follow-up measures via WMG-E web app  - Analyse data and prepare results for distribution to CFHN units, and preparation of peer-reviewed journal article | 02/2023 – 04/2023  (Month 21-23) |
| Implementation 2: Build capacity in NSW sites to commence rollout of the web app at scale | - Final training of CFHN units  - Ensure NSW Health is aware of findings  - Support the rollout of the tool in new sites | 04/2023 – 05/2023  (Month 23-24)  **Trial endpoint** |

**5.3 Data Source and Population**

**Randomised control trial (RCT) (Objective 1)**

At enrolment parents complete the consent form plus details about the child and parent (child’s name and date of birth, parent name and contact information). Parents on both conditions also complete the following instruments:

- Developmental surveillance (as per the Blue Book/Personal Health Record): Learn the Signs Act Early (LTSAE) [11], includes 8 ‘red flag’ questions with ‘yes’, ‘no’ and ‘not sure’ response categories, for children aged 2 months to 5 years. LTSAE is used to monitor children early development in the domains of social and emotional, language/communication; cognitive and movement/physical development. It takes about 3 minutes to complete.
- Unmet psychosocial needs: “WE CARE” questionnaire by Garg et al., 2007, consists of six (yes/no) items querying about: childcare, employment, homelessness, food security, education, and utilities. If caregivers answer “yes” to any question, a follow-up question appears asking parents whether they would like support in the nominated area (yes/no/maybe later).
- Mental health: Parental distress will be captured with the Kessler Psychological Distress Scale (K10) questionnaire. The K10 assesses psychological distress over the past 4 weeks by querying how often the responder felt nervous, hopeless, etc. on a five-point scale (All of the time, Most of the time, Some of the time, A little of the time, None of the time).

These instruments are administered again at the 6 and 12-month timepoints.

**Qualitative implementation evaluation study (Objective 2)**

We will conduct interviews at each site involving parents and CFHNs and other stakeholders. The parent interviews will involve parents of infant/toddler/pre-school aged children recruited as part of the research RCT via email invitations. The interviews with professionals will include CFHNs and other key stakeholders such as service providers, and policy representatives.

The interviews will measure the experience of using WMG-E using the Consolidated Framework for Implementation Research (CFIR) five implementation domains evaluating the acceptability, adoption, appropriateness, fidelity (use), coverage and sustainability of this equity focussed pathway by CFHNs. The CFIR constructs will be mapped onto the qualitative interviews and CFHNs will be asked about WMG-E acceptability, relevance, usefulness, and cultural appropriateness in their service and any barriers in using the program. Interview transcripts will be coded based on a preliminary coding scheme and analysed thematically with an iterative framework analysis using NVivo Pro 12 software.

The interviews for professionals will be centred around questions such as:

- Do you have any feedback about the referral guidelines/information provided by the WMG-E App?
- Does the use of a digital tool, such as WMG-E App, with the inclusion of a service navigator to link intervention families (participants) to the relevant services a feasible combination/approach for parents/caregivers with young children? Why/Why not?
- What do you perceive as the barriers/facilitators to using the WMG-E App?

The interviews for parents will be centred around questions such as:

- Do you have any feedback about the WMG-E App?
- Do you have any feedback about the information provided by the WMG-E App?
- What do you perceive as the potential barriers and facilitators to parents using the WMG-E App?
- What do you perceive as the potential barriers and facilitators to uptake of recommendations following identification of risk?

For intervention participants ONLY:

- Do you have any feedback about the service navigator you have been allocated to?
- Do you have any feedback about the information provided by your allocated service navigator?

A qualitative evaluation interview schedule, detailing the full interview questions, has been developed for this study and will be asked to parents/caregivers and health workers /professionals.

**5.4 Recruitment and Screening**

**Randomised Controlled Trial (RCT)**

For the RCT, two sites in NSW (Murrumbidgee and Fairfield/Liverpool/Bankstown LGAs) will ask families attending CFHS, refugee health services, supported playgroups, parenting groups, Karitane services, GP practices, special paediatric clinics, or Sustaining NSW families programs to complete the WMG-E web tool. Study flyer or poster will also be circulated within the listed local groups/services as well as the respective LHD/LGAs social media/communication network, so that families can choose to opt-in and access the WMG-E App (via a QR code or url link), should they wish to participate. Families will be randomized to either group upon accessing the web tool. Both groups will be screened for concerns and offered additional resources. In addition to this, the intervention group’s results will be emailed to the study coordinator, who will link families with the relevant services. Parents/caregivers will complete study consent, brief contact information and the three instruments described in section 5.3 online via the web app. These measures will also be made available in Arabic, Vietnamese, and Mandarin; the three most common non-English languages in NSW; where needed families will also be assisted by multicultural worker, refugee health nurse or other support workers.

**Qualitative implementation evaluation study**

For the qualitative interviews, parents of infants/toddlers/pre-schoolers and professionals/ policy makers/ stakeholders will be recruited by the research study coordinators to participate in approximately 30 to 60-minute interview to be held via Zoom/phone. Parents**:** Parents who participated in both the intervention and control groups will be recruited to interviews via email invitations inviting them to participate. They will also be sent the Participant Information Statement, and if they wish to find out more information or participate, they can email or call the research coordinators. Parents who chose not to participate in the research (i.e., dropped out of the research) will also be invited to participate in interviews via email. Professionals: Interviews will include CFHNs and other key stakeholders such as service providers and policy representatives. All CFHNs in participating sites as well as stakeholders will be invited to take part in qualitative interviews, via email invitations. Written informed consent to participate in the interviews (and audio-record the session) will be obtained from participants at the start of the interview.

**5.5 Inclusion Criteria**

**Randomised Controlled Trial (RCT)**

Parents/caregivers with a child aged 6 months to 3 years old attending CFHS, refugee health services, supported playgroups, parenting groups, Karitane services, GP practices, special paediatric clinics, or Sustaining NSW families programs, in either the Murrumbidgee LHD or Fairfield, Liverpool and Bankstown LGAs.

**Qualitative implementation evaluation study**

Parents/caregivers of children attending a CFHN Clinic, refugee health services, supported playgroups, parenting groups, Karitane services, GP practices, special paediatric clinics, or Sustaining NSW families programs that have participated in the WMG-E App research project for the full duration of the RCT in either sites are eligible to participate. This includes parents/caregivers who participated (along with those who did not participate for the entire trial duration) in the intervention. CFHNs working in the clinics, along with other stakeholders (policy makers/health professionals) are also eligible to participate in the interviews.

**5.6 Exclusion Criteria**

There are no exclusion criteria.

**5.7 Consent Process**

**Randomised Controlled Trial**

Parents/caregivers of children aged 6 months to 3 years old enrolled to attend the CFHS, refugee health service, supported playgroup, parenting groups, Karitane services, GP practices, special paediatric clinics, or Sustaining NSW families programs will be informed about the study by their CFHN/health worker/support group coordinator, who will email a web link to families. Study flyer or poster will also be circulated within the listed local groups/services as well as the respective LHD/LGA social media/communication network, so that families can choose to opt-in and access the WMG-E App (via a QR code or url link), should they wish to participate. The families will have the option to complete the consent form and the questions in the web-based app/link immediately. However, if the family has questions, they can respond to the email and the study team will subsequently call the family to explain the study and answer any questions the parents may have. The parents/caregivers will be given time to understand and consider their participation and for any questions or concerns to be fully addressed by staff. If parents/caregivers read the information and indicate an interest in participating, they will the complete the study consent form via the web tool. They will then complete the screening measures on the web tool. On completion of baseline questionnaires, a $20 Coles/Myer gift voucher will be given (either via postage or online) to parents as a gift for their participation. If a parent/caregiver does not wish to participate in the research study, they will continue to receive services as usual, that is, standard in-person developmental surveillance as provided by their CFHN, refugee health services, supported playgroups, parenting groups, Karitane services, GP practices, special paediatric clinics, or Sustaining NSW families programs.

The Participant Information Statement provides information about the reasons for the research, the risks/benefits, and the voluntary nature of the research. It also explains to participants that they decide to take part and later change their mind, that they are free to withdraw from the study at any stage and that this will not in any way affect the care of their child or family, from their CFHN or any other participation organisation.

**Qualitative implementation evaluation**

Parents and CFHNs and other stakeholders will be invited to participate in the interviews. The interviews will be advertised in these sites via flyers and emails to parents participating in the study. If participants wish to take part, interview sessions will be scheduled with the relevant researcher and held via phone/Zoom. At the time of the interview, the facilitator will explain the study participation and will obtain written information consent from all participants to participate in the interview, and to record the interview session via audio-recording. Participants will be informed that any identifying information will subsequently be redacted from the transcripts. The interviews will last 30 to 60 minutes. On completion of their interview, a $20 Coles/Myer gift voucher will be given (either via postage or online) to parents/caregivers ONLY (not to the service providers/healthcare professionals) as a gift for their additional participation. We would like to incentivize parents/caregivers for participating in the interviews (which is in addition to their main study time). Incentives for parents/caregivers are important, since we may be asking too much time/commitment from the participants to participate in the interviews (in addition to their main study time - the research trial).

The Participant Information Statement provides information about the reasons for the research, the risks/benefits, and the voluntary nature of the research. It also explains to participants that if they decide to take part and later change their mind, it will not possible to remove their responses from the audio-tape recording and the transcript, as the participants are de-identified and it will not be possible to identify which response is theirs.

**5.8 Study Procedures**

**Randomised Controlled Trial (RCT)**

For the RCT, two sites in NSW (Murrumbidgee LHD and Fairfield/Liverpool/Bankstown LGAs) with comparable population levels will be selected for participation in the study. Parents at each site who are attending CFHS, refugee health services, supported playgroups, parenting groups, Karitane services, GP practices, special paediatric clinics, or Sustaining NSW families programs will be invited to participate via email or flyer/poster distributed by the service on behalf of the study team. Participants may elect to participate immediately, or they can engage in a conversation with the study team (via phone or email) and participate at a later point in time. Parents/caregivers in both the intervention and care as usual conditions will be screened for psychosocial concerns and will be offered targeted resources. On completion of baseline, 6-month and 12-month time-point questionnaires, a $20 Coles/Myer gift voucher will be given (either via postage or online) to parents as a gift for their participation.

In addition to this, when parents allocated to the intervention arm will be linked to services by the study team. Children identified as being developmentally vulnerable or at-risk will be highlighted for follow-up by their local CFHS. For children identified as at risk, the Child and Family Health Nurse will contact the family to undertake further assessments and provide appropriate support and referral pathways for those children identified as having developmental concerns, as per usual care. Families identified as having financial difficulties or unmet psychosocial needs will be referred to support services (e.g. financial counsellor). Those who screen positive for mental health symptoms will be linked with a psychologist for further assessment and mental health first aid, along with brief interventions and referrals.

**The procedures are as follows:**

All participants:

1. When services choose to participate they are asked to contact all parents enrolled to attend their service and email them a link to the WMG-E web tool. The email contains the study teams contact details which families can use to contact the study team if they require more information.
2. Parents/caregivers of children will complete the consent form and baseline questionnaires online.
3. Parent/caregiver submit responses to questions and, if necessary, are provided with resources based on their responses (e.g. Uniting or Save the Children). Parents will also receive an email link to these resources. These results are also automatically sent to the study team.
4. On completion of baseline questionnaires, a $20 Coles/Myer gift voucher will be given (either via postage or online) to parents as a gift for their participation.

Families allocated to WMG-E+ CaU:

5. Following completion of baseline questionnaires, the study team will access the results from screening measures and a service navigator will be allocated to direct families to different care pathways depending on their presentation. This may include for example undertaking further assessments and providing support and referral pathways as deemed appropriate (as per usual care).

CaU /Control:

6. Following completing of the baseline questionnaires, participants will receive care as usual from the organizations they are already accessing services from. The study team will be sent results from the screening measures to screen responses. Participants who are allocated to the CaU group who are considered to be in “acute crisis” base on their questionnaire responses (i.e., K10 > 25 and/or at risk of homelessness, DV, loss of electricity, or no food) will automatically be considered RCT dropouts and will be contacted by a member of the research team who will be able to assist them in engaging with services.

All participants:

All participants will be sent follow-up emails at 6 and 12 months timepoints asking them to complete the same set of questionnaires via the web app again as part of their assessments. Families will receive a $20 Coles/Myer gift voucher as a gift for their time at completion of each timepoint surveys that are automatically emailed to consented parents.

**Qualitative implementation evaluation**

For the qualitative study, interviews will be conducted with parents and CFHNs/key stakeholders. The interview sessions will take 30 to 60 minutes. NVivo Qualitative software will be used as a data analysis tool to code the themes.

Parents/caregivers participating in the study will be invited to participate in the interviews via email. All service providers will be emailed and invited to participate in interviews as well. On completion of their interview, a $20 Coles/Myer gift voucher will be given (either via postage or online) to parents/caregivers ONLY (not to the service providers/healthcare professionals) as a gift for their additional participation. We would like to incentivize parents/caregivers for participating in the interviews (which is in addition to their main study time). Incentives for parents/caregivers are important, since we may be asking too much time/commitment from the participants to participate in the interviews (in addition to their main study time - the research trial).

**Data Analytic Plan**

The RCT will be registered with ANZCTR and will adhere to the CONSORT guidelines. Data analysis will examine the following:

**Research Question/Objective 1**.

Does WMG-E +CaU increase the wellbeing of families at 6 and 12 months post-intervention? We expect the WMG-E+ CaU will increase wellbeing (as defined by the combination of: parental mental health, child development, and unmet psychosocial needs) in the first 6 months, for both groups of parents (WMG-E + CaU and CaU). However, we expect families who are linked-in with services (i.e., WMG-E + CaU group) will show a significantly higher increase in wellbeing relative to the control group (i.e., CaU). These assumptions will be tested using a MANOVA and post-hoc comparisons.

**Objective 2. Evaluate/understand the experience of using WMG-E**

Interview transcripts for the interviews will be coded based on a preliminary coding scheme and analysed thematically with an iterative framework analysis using NVivo Pro 12 software.

**Objective 3. To measure the cost-effectiveness of WMG-E**

A ‘within-trial’ analysis will assess the cost effectiveness of WMG-E from the perspective of the health sector by using generalised estimating equations (GEE) to capture cost-effectiveness converting K-10 scores to health utilities using a mapping algorithm; and estimating affordability if the intervention is implemented routinely.

**Governance and management**

A steering committee consisting of the CI/AI team will meet bi-monthly along with project staff and representatives from key stake holders from across the state. This committee will be chaired by Dr Elisabeth Murphy, Senior Policy Advisor at NSW Health, who oversees Child and Family Health services at the state level, will serve as an Advisory Board for this project to oversee the operational aspects of the study.

5.9 **Randomisation**

Participants will be randomised automatically by the REDCap platform, assigning 50% of participants to each condition/group, stratified by site.

**5.10 Data Linkage (if applicable)**

Not applicable

# 6. TISSUE CoLLECTION/BIOBANKING (IF APPLICABLE)

Not applicable

**7. ETHICAL CONSIDERATIONS**

**7.1 Study Procedure Benefits**

The NSW State Blue Book/Personal Health Record (PHR) developmental surveillance program is *one of the critical preventative health programs* in the preschool period but it has come to a virtual standstill due to Child and Family Health Nurse (CFHN) clinics being mostly closed due to the COVID-19 pandemic. As a direct consequence of the significant reduction in CFHN clinics that are in operation, children are missing out on developmental checks, one of the critical preventative health programs in the preschool period. Similarly, the socioeconomic changes accompanying COVID-19 has meant that many families are in unstable positions and might be experiencing increased distress or psycho-social risk. To address this, we will implement and evaluate the WMG-E program as a support to the CFHN delivered in-person developmental clinics given the current gap in services within the state health CFNH clinic service due to COVID-19 related closure of clinics and redeployment of some of the staff to other tasks related to COVID-19. The WGM-E developmental assessment therefore uses the same questionnaire as what is in the Blue Book (the LTSAE) which is what nurses would ask parents to complete if they were to conduct a face-to-face assessment.

Participants in the RCT will be informed that by participating in this study they may be more aware of the signs and symptoms of developmental disorders and delay, regardless of whether their child is identified as being at risk of developmental disorders. They will also be informed that this information and knowledge may be of benefit to them in future in their ongoing monitoring of their child’s development or in dealings with other children, friends or family members. Participants in the qualitative study will be informed that there will not be any benefits to participation in the in-depth interviews.

**7.2	Study Procedure Risks**

*Potential risk*: There are no major risks to participants associated with participation in this trial. However, as parents/caregivers participating in the RCT will complete measures about their child’s development, their own mental health, and their psychosocial needs, it is possible that answering these questions may cause distress to participants, especially if parents/caregivers are told their child may have a developmental delay or that they themselves may have a undiagnosed mental health condition. Similarly, for the parent participants in the interviews, answering questions about developmental surveillance, child development, and their psychosocial and psychological needs may cause participants to experience distress.

*Risk likelihood:* Possible

*Risk impact*: Moderate

*Risk mitigation strategy*: To address the risk of potential distress to participants in the RCT, the following steps will be taken:

1. The study team will be able to provide extra time with the family to support them regarding their distress and concerns following completion of questionnaires at each time point and will be able to direct them to CFHS if they request assistance.
2. Contact details for a range of mental health support services and local services to support child development are provided to participants in the Participant Information Statement.
3. Participants are told in the Participant Information Statement that they can stop participating at any time if they experience any feelings of distress.

Similarly, for the interviews, participants are told that they can stop participating at any time and contact details for a range of mental health support services and local services to support child development are provided to participants.

*Generalizability of the data:* The data gathered from this study is expected to be generalizable to multicultural and rural communities, the two demographic groups targeted by this plan. In order to ensure the data is generalizable it will be important to seek ways of engaging with families outside of CFHS – which are currently poorly attended in NSW. This is why the addition of refugee health services and supported playgroups was an important consideration to the current study, as it would ensure that other parents, beyond those that are highly motivated, get a chance to take part in the study.

**7.3 Confidentiality and Privacy**

**Randomized Controlled Trial (RCT)**

Participant data and information will be kept private and confidential. Data will be de-identified at the point of entry into the study. A unique ID number will be generated when participants give consent to participate and enter their data into the WMG web tool. Family identifying information and IDs will be classified as identifying information in the REDCap system and will be subject to increased protection measures. All files on the UNSW REDCap are password protected and hosted in Australian servers. All operational trial data will be stored electronically on SWSLHD password-protected computers and only accessible to a small number of Chief Investigators.

All reports will ensure that any data or information reported will not be identifiable. Only aggregate data will be presented.

**Qualitative implementation evaluation**

All participants in the interview will be assured that any potentially identifiable information revealed during the interviews will be redacted in the transcripts. This will ensure that no individuals can be identified through this process. The de-identified transcripts and coded data will be held on SWSLHD password-protected computers and only available to research staff.

**7.4 Data Storage and Record Retention**

The survey data will be held securely in UNSW REDCap servers that are password protected. All operational trial data (e.g. hard copies of flyers, ethics documentation, etc) will be stored electronically on SWSLHD password-protected network drives and only accessible to a small number of Chief Investigators. No external personnel will have access to the data.

For data collected via the WMG web tool, the survey tool REDCap will be used to collect data, and the built-in audit trail will allow administrators to be able to determine all the activity and all the data viewed or modified by any given user.

The electronic data and paper files will be kept for 25 years, and the database will be deleted at the end of this time.

**7.5 Data Monitoring**

The study may be audited by the South Western Sydney LHD ethics committee and inspected by government regulatory authorities. Any source information and other study files will be made accessible at all study sites (South Western Sydney LHD and Murrumbidgee LHD) at the time of auditing and inspection during the course of the study and after the completion of the study.

# 8. SAFETY REPORTING (IF APPLICABLE)

Since this study does not involve any investigational therapeutic goods or devices, all adverse events and other unintended effects of trial intervention or conduct will be reported to the relevant authorities as per the principles of the NHMRC National Statement on Ethical Conduct in Human Research guidelines. This information will also be reported to the DMC and included as part of the project’s annual progress report to the relevant ethics committee.

# 9. Data Safety and Monitoring Board (Clinical Trials only)

A small, independent Data Monitoring Committee (DMC) will be established and will meet once every six months to monitor the quality of the trial data and safety of research participants. DMC membership will consists of experts in clinical trial conduct in health services research, statistics, mental health and child development. DMC will monitor blinded response variables and safety outcomes for early dramatic benefits or potential adverse events and provide reports to the investigators on recommendations to continue or temporarily halt recruitment to the study. The committee will be governed by a charter that will outline their responsibilities, procedures and confidentiality.

# 10. Early Termination (IF APPLICABLE)

Not applicable

# 11. BLINDING AND UNBLINDING (if applicable)

Participants are not blind to the randomisation.

**12. CONFLICT OF INTEREST**

There is no potential duality or conflict of interest for investigators arising from their role in this study.

**13. FUNDING**

This project is funded by the NSW Ministry of Health COVID-19 Research Grants Round 2.

**14. RESEARCH OUTCOMES**

The outcomes of the research include:

Publication of study findings in peer-reviewed journals.

The findings will be presented at national and international workshops and conferences.

There are no plans for sharing the data or secondary analyses of the data beyond those described in this application.

Participants in the interviews and the RCT will be asked at the time of providing consent to participate whether they wish to receive a copy of the results of the trial. Participants can elect to receive or not receive the results and provide their email address or postal address to receive the results.

**15. REFERENCES**

1. Centre for Community Child Health and Telethon Institute for Child Health Research, *A Snapshot of Early Childhood Development in Australia - AEDI National Report 2009*, 2009: Canberra.
2. Australian Institute of Health and Welfare, *Headline Indicators for Children's Health Development and Wellbeing*, 2011: Canberra.
3. Grantham-McGregor, S., et al. *The Lancet*, 2007. **369**(9555): p. 60-70.
4. Walker, S.P., et al., *The Lancet*, 2011. **378**(9799): p. 1325-1338.
5. Eapen, V., et al. *BMC Pediatrics*, 2014. **14**(1): p. 234.
6. Woolfenden, S., et al. *BMC Pediatrics*, 2016. **16**(1): p. 46.
7. Overs, B., et al., *Child: Care, Health & Development*, 2016.
8. Chandra, M., et al. *BMJ Open*, 2016. **6**(10): p. e012342.
9. Isaacs, J.B., *Cost effective investments in children*. 2007, Washington DC: Brookings Inst.
10. Heckman, J.J., et al., *The productivity argument for investing in young children*, 2007, National Bureau of Economic Research.
11. Raspa, M., et al, *Journal of Developmental and Behavioral Pediatrics 2015*, ***36***(8), p.575.
12. Murray, C., et al BMC Medicine, 2010. 10 (1); p 61, [DOI: 10.1186/1472-6947-10-61](https://doi.org/10.1186/1472-6947-10-61)
13. Fixsen, D.L. ***Implementation Research: A Synthesis of the Literature*** National Implementation Research Network, University of Florida, Tampa, FL (2005)
14. Weiner, B. J., Lewis, C. C., Stanick, C., Powell, B. J., Dorsey, C. N., Clary, A. S., Boynton, M. H., & Halko, H. (2017). Psychometric assessment of three newly developed implementation outcome measures. Implementation Science, 12(108), 1-12. doi: 10.1186/s13012-017-0635-3.
