# Supplementary material for: Determinants of unmet social needs and the role of parental mental health in families from multicultural and regional/rural communities of Australia
Source: PLoS One. 2026 Aug 11;21(8):e0332560. doi: 10.1371/journal.pone.0332560 (PMC13460624; doi:10.1371/journal.pone.0332560)
Supplement: Supplementary file 3 [file pone.0332560.s003.docx]

**Response letter**

*We thank the editorial staff for the initial review. We would like to note that the trial outcomes manuscript is currently under review in another journal. While that article focuses on reporting the primary and secondary outcomes as outlined in the study protocol, the present manuscript takes a complementary perspective by examining the role of sociodemographic risk factors and parental mental health in unmet needs among families from multicultural and regional/rural communities in Australia.*

1. PLOS ONE requires that all clinical trials are registered in an appropriate registry (the WHO list of approved registries is at https://www.who.int/clinical-trials-registry-platform/network/primary-registries" https://www.who.int/clinical-trials-registry-platform/network/primary-registries and more information on trial registration is at <http://www.icmje.org/about-icmje/faqs/clinical-trials-registration/>).

Please state the name of the registry and the registration number (e.g. ISRCTN or ClinicalTrials.gov) in the submission data and on the title page of your manuscript.

***Author response:*** *We have added this information (Page 3).*

a) Please provide the complete date range for participant recruitment and follow-up in the methods section of your manuscript.

***Author response:*** *We have added this information in the methods section (Page 8).*

b) If you have not yet registered your trial in an appropriate registry, we now require you to do so and will need confirmation of the trial registry number before we can pass your paper to the next stage of review. Please include in the Methods section of your paper your reasons for not registering this study before enrolment of participants started. Please confirm that all related trials are registered by stating: “The authors confirm that all ongoing and related trials for this drug/intervention are registered”.

***Author response:*** *We have added this information in the methods section (Page 7).*

2. Please note that you must upload a completed CONSORT flowchart as figure 1 of your manuscript. Blank copies of this document and information regarding CONSORT can be found via the following link: (<https://www.equator-network.org/reporting-guidelines/consort/>).

***Author response:*** *We have added CONSORT flowchart as figure 1.*

3. Please note that you must upload a completed CONSORT checklist as a supporting information file. Blank copies of this document and information regarding CONSORT can be found via the following link: (<https://www.equator-network.org/reporting-guidelines/consort/>). If your clinical trial uses a non-randomized design, you may wish to submit a TREND checklist (<https://stacks.cdc.gov/view/cdc/149677>), in place of the CONSORT checklist.

***Author response:*** *As noted in our initial comment, this manuscript reports a secondary outcome analysis of the trial, for which we had originally followed the STROBE guidelines. In response to the request, we have now included the CONSORT checklist as a supporting information file. To avoid unnecessary repetition and to maintain alignment with the objectives of this study, we have not reiterated details on randomisation, blinding, intervention, or sample size calculation; instead, we have directed readers to the published protocol, which is appropriately referenced.*

4. Please upload a copy of your trial study protocol as a supporting information file. By the study protocol, we mean the complete and detailed plan for the conduct and analysis of the trial that the ethics committee approved before the trial began. Please send this in the original language. If this is in a language other than English, please also provide a translation. Please detail any deviations from this study protocol in the Methods section of your manuscript. Your study protocol will be made available to the editors and reviewers, and will be published as supporting information with your manuscript if accepted for publication. (If you do not agree to this, we will not be able to publish your manuscript). If you have formally published a study protocol for your trial in a journal then you should cite this in your manuscript, but you still need to send us the original document.

***Author response:*** *We have added the study protocol as a supporting information file and cited the published protocol in the methods section. The protocol outlines analyses of the primary and secondary outcomes; however, this manuscript extends those findings by examining the influence of sociodemographic risk factors and parental mental health on unmet needs among families from multicultural and regional/rural communities in Australia. We consider this a complementary perspective consistent with the study aims, rather than a deviation from the original protocol.*

5. The in-house editorial staff feels that your study meets the World Health Organization definition of a clinical trial because it is a prospective study in which participants were assigned to a service navigator intervention to investigate the effects on psychosocial needs, mental health outcomes, and developmental outcomes, as detailed in the trial registration page: <https://www.anzctr.org.au/Trial/Registration/TrialReview.aspx?id=381790&isReview=true>. You have indicated that this is a secondary analysis of RCT data. However the analysis presented in this study matches the primary outcomes listed in the RCT registration page and it includes an analysis of the main intervention's effects. Additionally, the trial's results have not been published elsewhere, to our knowledge. In light of this, please change your manuscript’s article type to ‘Clinical Trial’ when you resubmit your manuscript.

***Author response:*** *We thank the editorial staff for the initial review. We will change the manuscript’s article type to ‘Clinical Trial’. However, we would like to note that the trial outcomes manuscript is currently under review in another journal. While that article focuses on reporting the primary and secondary outcomes as outlined in the study protocol, the present manuscript takes a complementary perspective by examining the role of sociodemographic risk factors and parental mental health in unmet needs among families from multicultural and regional/rural communities in Australia.*

Additionally, please clarify whether recruitment began at the same time at each study site. If not, please report in the Methods section the day, month and year of the start and end of the recruitment period for each study site. Please note that if this information is not included when your manuscript is resubmitted, it may be rejected"

***Author response:*** *We have added this information in the methods section (Page 8).*
