# Supplementary material for: Determinants of unmet social needs and the role of parental mental health in families from multicultural and regional/rural communities of Australia
Source: PLoS One. 2026 Aug 11;21(8):e0332560. doi: 10.1371/journal.pone.0332560 (PMC13460624; doi:10.1371/journal.pone.0332560)
Supplement: Supplementary file 4 [file pone.0332560.s004.docx]

**Point-by-point response to Reviewers’ comments**

Dear Editor,

We thank you and the reviewers for your time in reviewing our paper and providing valuable comments that have helped to improve the current version. We have carefully considered the comments and responded to all the items. We hope the manuscript after careful revisions meet your high standards. Below we provide the point-by-point responses. All modifications in the manuscript have been highlighted using tracked changes.

Editor’s comments

1. Please ensure that your manuscript meets PLOS ONE's style requirements, including those for file naming. The PLOS ONE style templates can be found at https://journals.plos.org/plosone/s/file?id=wjVg/PLOSOne_formatting_sample_main_body.pdf and https://journals.plos.org/plosone/s/file?id=ba62/PLOSOne_formatting_sample_title_authors_affiliations.pdf

***Author response:*** We have carefully formatted the manuscript as per PLOS ONE's style requirements.

1. Your ethics statement should only appear in the Methods section of your manuscript. If your ethics statement is written in any section besides the Methods, please move it to the Methods section and delete it from any other section. Please ensure that your ethics statement is included in your manuscript, as the ethics statement entered into the online submission form will not be published alongside your manuscript.

***Author response:*** We have now moved the ethics statement to the methods section as requested (See Page 7, Lines 128-134).

2. We note that you have indicated that there are restrictions to data sharing for this study. For studies involving human research participant data or other sensitive data, we encourage authors to share de-identified or anonymized data. However, when data cannot be publicly shared for ethical reasons, we allow authors to make their data sets available upon request. For information on unacceptable data access restrictions, please see <http://journals.plos.org/plosone/s/data-availability#loc-unacceptable-data-access-restrictions>.

***Author response:*** We have revised the data sharing statement in the manuscript (See Page 18, Lines 371-380).

3. Before we proceed with your manuscript, please address the following prompts:
a) If there are ethical or legal restrictions on sharing a de-identified data set, please explain them in detail (e.g., data contain potentially identifying or sensitive patient information, data are owned by a third-party organization, etc.) and who has imposed them (e.g., a Research Ethics Committee or Institutional Review Board, etc.). Please also provide contact information for a data access committee, ethics committee, or other institutional body to which data requests may be sent.

b) If there are no restrictions, please upload the minimal anonymized data set necessary to replicate your study findings to a stable, public repository and provide us with the relevant URLs, DOIs, or accession numbers. Please see <http://www.bmj.com/content/340/bmj.c181.long> for guidelines on how to de-identify and prepare clinical data for publication. For a list of recommended repositories, please see <https://journals.plos.org/plosone/s/recommended-repositories>. You also have the option of uploading the data as Supporting Information files, but we would recommend depositing data directly to a data repository if possible.

Please update your Data Availability statement in the submission form accordingly.

***Author response:*** We have revised the data sharing statement in the manuscript (See Page 18, Lines 371-380).

4. In the online submission form, you indicated that data are available upon reasonable request. Data collected for this study will be shared upon reasonable request to the corresponding author ([v.eapen@unsw.edu.au](mailto:v.eapen@unsw.edu.au))

All PLOS journals now require all data underlying the findings described in their manuscript to be freely available to other researchers, either 1. In a public repository, 2. Within the manuscript itself, or 3. Uploaded as supplementary information.
This policy applies to all data except where public deposition would breach compliance with the protocol approved by your research ethics board. If your data cannot be made publicly available for ethical or legal reasons (e.g., public availability would compromise patient privacy), please explain your reasons on resubmission and your exemption request will be escalated for approval.

***Author response:*** We have revised the data sharing statement in the manuscript (See Page 18, Lines 371-380).

5. We note that the grant information you provided in the ‘Funding Information’ and ‘Financial Disclosure’ sections do not match.

***Author response:*** We have now updated this information to match in both sections.

6. When you resubmit, please ensure that you provide the correct grant numbers for the awards you received for your study in the ‘Funding Information’ section.

***Author response:*** We have now updated this information for both NSW Health COVID-19 Research Grants Round 2 and National Health and Medical Research Council (NHMRC) Investigator Grant. We have included the application number for the NSW Health COVID-19 Research Grants Round 2 as there was no specific grant identifier number provided (See Pages 18-19, Lines 392-402).

7. Thank you for stating the following financial disclosure:

This study was supported through the NSW Health COVID-19 Research Grants Round 2, following an independent peer-review process, and delivered in partnership with the University of New South Wales, South Western Sydney Local Health District, Murrumbidgee Local Health District, NSW Ministry of Health, Sydney Children’s Hospital Randwick, Western Sydney University, Ingham Institute for Applied Medical Research, Black Dog Institute, Uniting, and Karitane. The funding body had no role in the study design, data collection, analysis, interpretation, or in the writing of this manuscript. VE is supported by National Health and Medical Research Council (NHMRC) Investigator Grant #2033610

Please state what role the funders took in the study. If the funders had no role, please state: "The funders had no role in study design, data collection and analysis, decision to publish, or preparation of the manuscript."

If this statement is not correct you must amend it as needed.

Please include this amended Role of Funder statement in your cover letter; we will change the online submission form on your behalf.

***Author response:*** We have now added the above sentence about the role of funders in the manuscript (See Pages 19, Lines 399-402) and the cover letter.

8. Please amend either the title on the online submission form (via Edit Submission) or the title in the manuscript so that they are identical.

***Author response:*** We have now updated this information to match in both sections.

9. Please amend the manuscript submission data (via Edit Submission) to include author Blaise Di Mento.

***Author response:*** We have now updated this information.

10. Please include a caption for figure 1.

***Author response:*** We have now included a caption for figure 1 (See Page 34).

11. We note that there is identifying data in the Supporting Information file <Detailed Study protocol.docx>. Due to the inclusion of these potentially identifying data, we have removed this file from your file inventory. Prior to sharing human research participant data, authors should consult with an ethics committee to ensure data are shared in accordance with participant consent and all applicable local laws.

Data sharing should never compromise participant privacy. It is therefore not appropriate to publicly share personally identifiable data on human research participants. The following are examples of data that should not be shared:

-Name, initials, physical address

-Ages more specific than whole numbers

-Internet protocol (IP) address

-Specific dates (birth dates, death dates, examination dates, etc.)

-Contact information such as phone number or email address

-Location data

-ID numbers that seem specific (long numbers, include initials, titled “Hospital ID”) rather than random (small numbers in numerical order)

Data that are not directly identifying may also be inappropriate to share, as in combination they can become identifying. For example, data collected from a small group of participants, vulnerable populations, or private groups should not be shared if they involve indirect identifiers (such as sex, ethnicity, location, etc.) that may risk the identification of study participants.

Additional guidance on preparing raw data for publication can be found in our Data Policy (<https://journals.plos.org/plosone/s/data-availability#loc-human-research-participant-data-and-other-sensitive-data>) and in the following article: <http://www.bmj.com/content/340/bmj.c181.long>.

Please remove or anonymize all personal information (<specific identifying information in file to be removed>), ensure that the data shared are in accordance with participant consent, and re-upload a fully anonymized data set. Please note that spreadsheet columns with personal information must be removed and not hidden as all hidden columns will appear in the published file.

***Author response:*** We thank the Editorial team for this feedback. We confirm that the detailed study protocol document only contained names of the investigatory team and not research participants’ data. We have now removed the details of the investigatory team as well.

12. [Please include captions for your Supporting Information files at the end of your manuscript, and update any in-text citations to match accordingly. Please see our Supporting Information guidelines for more information: http://journals.plos.org/plosone/s/supporting-information.](http://track.editorialmanager.com/CL0/http:%2F%2Fjournals.plos.org%2Fplosone%2Fs%2Fsupporting-information./1/010f0199f024e332-cdfa8289-8623-4c62-a371-ea4416312b5f-000000/m16xJM2wHs9g33Hs0Z5ctTCPIMWqxAttmfPpzHgwDxw=233)

***Author response:*** We have now added the captions for the supporting information files in text and at the end of the manuscript (See Page 34).

13. If the reviewer comments include a recommendation to cite specific previously published works, please review and evaluate these publications to determine whether they are relevant and should be cited. There is no requirement to cite these works unless the editor has indicated otherwise.

***Author response:*** We thank the Editorial team for this information.

**Reviewers' comments:**

Reviewer #1:

Dear Editor,

I write to submit my review report on the manuscript titled “Determinants of unmet social needs and the role of parental mental health in families from multicultural and regional/rural communities of Australia.” The study explored the determinants of social care needs and the associated clinical characteristics such as parental mental health among families from multicultural and regional/rural communities of Australia

Comments

Methods
1.The authors stated that the presence of unmet needs was dichotomized into two categories: no unmet needs (WE-CARE score = 0) and one or more unmet needs (WE-CARE score ≥ 1). They then fitted a binary logistic regression model to assess the determinants. Sensitivity analyses must be conducted when the outcome is treated as a count (i.e., the number of unmet needs) and modeled using either a Poisson or a negative binomial regression model. You lose so much information if we re-categorize count outcomes. For instance, 2, 3, 4, 5, 6 or higher number of unmet needs are still classified as 1 and logistic regression will not help isolate the differences in the number of unmet needs

***Author response:*** Thank you for your feedback. To conform with reporting standards for cross-sectional studies, we have revised the statistical methods. The data was overdispersed, hence univariable and multivariable analyses were conducted with negative binomial regression. We check for overdispersion using the ‘overdisp’ module in Stata (See Methods section – Page 4, Lines 63-66 and Pages 9-10, Lines 183-196).

**Modified sections:** *“Univariable negative binomial regression analysis was used to determine the factors independently associated with increased rate of unmet needs, where unmet needs were treated as count data for each unmet need. Factors included sociodemographic, sociocultural, and clinical indicators where unmet social needs at baseline (model 1), 6 months (model 2), and 12 months (model 3) were the respective outcomes. The analyses repeated the above three models in a multivariable negative binomial regression analysis with all the above exposures at once for each of the models. Negative binomial regression was used due to overdispersion seen between the outcome and each of the above factors. All analyses were undertaken in Stata v19 (StataCorp. 2025. Stata Statistical Software: Release 19. College Station, TX: StataCorp LLC.)”*

Please also see revised analysis in Tables 2 and 3 (Pages 27-34), results section (Pages 11-12, Lines 215-257) and the discussion section (Page 14, 286-293).

2.For a baseline data, the design is basically cross-sectional and the preferred effect size of interest should be prevalence ratio not odds ratio(Barros & Hirakata, 2003). You can generate prevalence ratio for binary outcomes using log-binomial models, modified Poisson (Poisson with robust standard errors), and Cox-proportional models with robust variance estimation(Barros & Hirakata, 2003; Coutinho, Scazufca, & Menezes, 2008; Martinez et al., 2017; Talbot, Mésidor, Chiu, Simard, & Sirois, 2022). Usually, researchers think that you can only model a binary outcome with only probit and logistic regression models and Poisson is for modeling only count outcomes. Poisson and Negative binomial regression models with robust standard errors can be used to model both count and binary outcomes and here is the list of references in the medical literature where different authors compare Poisson with robust standard error, log-binomial models, and logistic regression models, etc. when they analyzed BINARY OUTCOMES using cross-sectional data. Conducting a sensitivity analysis that reports the odds ratio and prevalence ratio from a binary outcome may enhance the paper by treating the outcome measure as a count

References
Bastos, L. S., Oliveira, R. D. V. C. D., & Velasque, L. D. S. (2015). Obtaining adjusted prevalence ratios from logistic regression models in cross-sectional studies. Cadernos de saude publica, 31, 487-495.

Gnardellis, C., Notara, V., Papadakaki, M., Gialamas, V., & Chliaoutakis, J. (2022). Overestimation of relative risk and prevalence ratio: misuse of logistic modeling. Diagnostics, 12(11), 2851

Coutinho, L., Scazufca, M., & Menezes, P. R. (2008). Methods for estimating prevalence ratios in cross-sectional studies. Revista de saude publica, 42, 992-998.
Barros, A. J., & Hirakata, V. N. (2003). Alternatives for logistic regression in cross-sectional studies: an empirical comparison of models that directly estimate the prevalence ratio. BMC medical research methodology, 3(1), 1-13.

Wolkewitz, M., Bruckner, T., & Schumacher, M. (2007). Accurate variance estimation for prevalence ratios. Methods of Information in Medicine, 46(05), 567-571.

***Author response:*** Thank you for your feedback. To conform with reporting standards for cross-sectional studies, we have revised the statistical methods. The data was overdispersed, hence univariable and multivariable analyses were conducted with negative binomial regression. We check for overdispersion using the ‘overdisp’ module in Stata (See Methods section – Page 4, Lines 63-66 and Pages 9-10, Lines 183-196).

**Modified sections:** *“Univariable negative binomial regression analysis was used to determine the factors independently associated with increased rate of unmet needs, where unmet needs were treated as count data for each unmet need. Factors included sociodemographic, sociocultural, and clinical indicators where unmet social needs at baseline (model 1), 6 months (model 2), and 12 months (model 3) were the respective outcomes. The analyses repeated the above three models in a multivariable negative binomial regression analysis with all the above exposures at once for each of the models. Negative binomial regression was used due to overdispersion seen between the outcome and each of the above factors. All analyses were undertaken in Stata v19 (StataCorp. 2025. Stata Statistical Software: Release 19. College Station, TX: StataCorp LLC.)”*

Please also see revised analysis in Tables 2 and 3 (Pages 27-34), results section (Pages 11-12, Lines 215-257) and the discussion section (Page 14, 286-293).

3.The authors indicated that univariate linear regression analysis was used to determine the factors independently associated with the risk of unmet needs. Why linear regression when the outcome measure is binary? Is it univariate logistic regression or linear regression, especially when you reported an odds ratio? Clarify

***Author response:*** We thank the reviewer for noting this error. We have now replaced the term “linear” to negative binomial regression (Pages 9-10, Lines 183-196).

**Modified sections: “***Univariable negative binomial regression analysis was used to determine the factors independently associated with increased rate of unmet needs, where unmet needs were treated as count data for each unmet need.”*

4.The statement “the multivariate binary logistic regression models” should rather be multivariable binary logistic regression model. Multivariate mean modelling two or more dependent variables (outcome measures) simultaneously. Kindly revise

***Author response:*** We have now revised the “multivariate binary logistic regression models” to multivariable negative binomial regression model (Pages 9-10, Lines 183-196).

**Modified sections:** *“The analyses repeated the above three models in a multivariable negative binomial regression analysis with all the above exposures at once for each of the models. Negative binomial regression was used due to overdispersion seen between the outcome and each of the above factors.”*

5.Put a footnote of the meaning of n (%) or M(SD) under Table 1

***Author response:*** We have added the footnote under Table 1 (See Pages 24-26).

6. What was the overall prevalence of unmet needs and the corresponding 95% confidence interval at baseline and how does it vary across the background characteristics. This important information is missing in the manuscript

***Author response:*** Thank you for your feedback. We have now provided row percentages for one or more unmet needs and no unmet needs for each demographic characteristic. We believe that writing 95% confidence intervals in a descriptive table of summary statistics is inappropriate use of statistical inference due to multiple testing concerns (See Pages 24-26).
