# Supplementary material for: Determinants of unmet social needs and the role of parental mental health in families from multicultural and regional/rural communities of Australia
Source: PLoS One. 2026 Aug 11;21(8):e0332560. doi: 10.1371/journal.pone.0332560 (PMC13460624; doi:10.1371/journal.pone.0332560)
Supplement: Supplementary file 5 [file pone.0332560.s005.docx]

**Point-by-point response to Reviewers’ comments**

Dear Editor,

We thank you and the reviewers for your time in reviewing our paper and providing valuable comments that have helped to improve the current version. We have carefully considered the comments and responded to all the items. We hope the manuscript after careful revisions meet your high standards. Below we provide the point-by-point responses. All modifications in the manuscript have been highlighted using tracked changes.

**Reviewers' comments:**

Reviewer #1:

1. Almost all my comments have been addressed adequately except this one. "Author response: Thank you for your feedback. We have now provided row percentages for one or more unmet needs and no unmet needs for each demographic characteristic. We believe that writing 95% confidence intervals in a descriptive table of summary statistics is inappropriate use of statistical inference due to multiple testing concerns (See Pages 24-26)". Unfortunately that is not the case as this has nothing to do with testing the difference. Is purely estimation. No p-values. Point estimates alone does not fully tell us what happens in the population. Kindly estimate the CIs.

***Author response:*** *We thank the reviewer for the feedback. We agree that 95% confidence intervals provide information on the precision of descriptive estimates and are not related to hypothesis testing. Accordingly, we have now estimated and reported 95% confidence intervals for the row percentages in the descriptive table (Table 1).*

***Modifications made:*** *See Table 1.*
