# Supplementary material for: Determinants of unmet social needs and the role of parental mental health in families from multicultural and regional/rural communities of Australia
Source: PLoS One. 2026 Aug 11;21(8):e0332560. doi: 10.1371/journal.pone.0332560 (PMC13460624; doi:10.1371/journal.pone.0332560)
Supplement: Supplementary file 6 [file pone.0332560.s006.docx]

**Point-by-point response to Reviewers’ comments**

Dear Editor,

We thank you and the reviewers for your time in reviewing our paper and providing valuable comments that have helped to improve the current version. We have carefully considered the comments and responded to all the items. We hope the manuscript after careful revisions meet your high standards. Below we provide the point-by-point responses. All modifications in the manuscript have been highlighted using tracked changes.

**Reviewers' comments:**

**Reviewer #1**

1. Delete the third column of Table 1. I was referring to row percentages and corresponding 95% confidence interval for the outcome measure for each of the background characteristics. You have now provided that in column 5 and 7, perfect. Just delete the third column of Table 1. Ideally you should have one table for the background characteristics and another Table for the prevalence (row percentages) and confidence interval estimates for each background characteristics but you lumped them in Table 1 with the pure descriptive statistics. That was why I said in my previous review to estimate confidence interval for the outcome measure for each background characteristics. Once you delete the third column of Table 1, everything will fall in place. Note that prevalence (row percentages) is referring to the outcome measure. For instance, among those who experience premature birth, what percentage had say one or more unmet needs, the answer is 61.0% [95%CI: 54.9-66.5]. This is what I was referring to. You must categorize the continuous variables like age into meaningful and relevant policy-based age categories and estimate the prevalence of experiencing one or more unmet needs across each category. This applies to other continuous variables in Table 1. Another way to do this is to keep Table 1 but only column 1 and 2 which is purely descriptive and delete other remaining columns. Then you create table 2 and titled that Table " Prevalence of one or more unmet needs by background characteristics". So for Table 2, you will have two columns, Column 1 is the background characteristics and column 2 is Prevalence [95% CI]. This is very informative and will improve the manuscript

***Author response:*** *We thank the reviewer for this feedback. We have revised the tables accordingly. Specifically, we deleted the third column from Table 1 and retained Table 1 as a descriptive summary of the sample characteristics. As suggested, we have also created a new Table 2 titled “Prevalence of One or More Unmet Needs by Background Characteristics,” which presents the row prevalence estimates with corresponding 95% confidence intervals across each background characteristic category. In addition, to provide a more complete representation of the outcome distribution, Table 2 now includes both participants with one or more unmet needs and those with no unmet needs.*

*We have also categorised continuous variables into meaningful policy-relevant categories to improve interpretability and alignment as suggested.*

***Modifications made:*** *See Tables 1 and 2.*

**Reviewer #2**

The statistical section is generally well structured and appropriate for a public health study, but there are several strengths and areas that could be improved for clarity, rigor, and reporting quality. The study clearly defines the primary outcome as: Unmet social care needs, Measured using the WE CARE survey and Treated as a count variable. This is statistically appropriate because unmet needs are naturally count data. The author’s state: As the data were over dispersed, univariable and multivariable negative binomial regression models were applied…. This is a strong methodological choice for count data. The study used Univariable analysis and Multivariable negative binomial regression which helps Identify crude associations and adjust for confounding variables. And good practice in epidemiological and clinical studies.

Statistical Areas for Improvement

a) Randomized Controlled Trial Design Not Fully Reflected in Analysis. The study is described as secondary analysis of a randomized controlled trial, however, the statistical section does not explain: Whether treatment group allocation was adjusted for, whether intervention effects were controlled and whether clustering/repeated measures were considered. This is important because repeated observations over time may violate independence assumptions. If possible better approach or if the authors could consider: Mixed-effects negative binomial regression, Generalized Estimating Equations (GEE) since measurements were taken repeatedly over time.

***Author response:*** *We thank the reviewer for this feedback. We agree that the longitudinal nature of the data requires consideration of within-subject correlation arising from repeated measurements over time. To address this, we conducted a Generalized Estimating Equations (GEE) negative binomial regression analysis in addition to the multivariable negative binomial regression model. The GEE approach accounts for the non-independence of repeated observations and provides population-averaged estimates while accommodating the overdispersed count nature of the outcome. We have also removed the univariable regression tables and results to avoid too many tables.*

***Modifications made:***

*Methods section – “To assess the robustness of these findings and to account for repeated measurements over time, a Generalised Estimating Equations (GEE) negative binomial model with an exchangeable correlation structure was additionally fitted. This model incorporated all three time points (baseline, 6 months, and 12 months) and accounted for within‑participant clustering. Treatment group allocation (intervention vs control) was included as a covariate to reflect the underlying randomised controlled trial design.” (Page 10, lines 196-201).*

*Results section – “To assess the robustness of these findings and account for repeated measurements over time, a GEE negative binomial model was fitted (Table 4). The GEE model incorporated all three time points simultaneously and adjusted for within participant clustering. The results were highly consistent with the standalone negative binomial models. Significant predictors in the GEE model included parental psychological distress, number of child developmental concerns, CALD background, lower parental education, marital status, and parental age. The intervention group showed a significantly lower rate of unmet needs (AIRR 0.75, 95% CI: 0.60, –0.95), consistent with the direction of effects observed in the original models.” (Page 13, lines 260-267).*

*Discussion section – “Additionally, findings of the GEE model further suggest that these factors may have a sustained influence on families’ unmet social needs over time, rather than reflecting challenges at baseline.” (Page 14, lines 287-289).*

*See Table 4 (Page 36).*

b) Missing Model Diagnostics: If the article does discuss Goodness-of-fit, Residual analysis, multicollinearity and influential observations. And I will recommended diagnostic contains Variance Inflation Factor (VIF), Residual plots, AIC/BIC and Pearson residuals

***Author response:*** *We have now added model diagnostics such as variance-to-mean ratio, Variance Inflation Factors (VIFs), Pearson residuals and fitted-value plots.*

***Modifications made:***

*Methods section – “Model diagnostics were conducted to assess the suitability of the count regression models. Overdispersion was evaluated using the variance-to-mean ratio, multicollinearity was assessed using Variance Inflation Factors (VIFs), and model fit was examined through inspection of Pearson residuals and fitted-value plots.” (Page 10, lines 203-206).*

*Results section – “Diagnostic evaluation supported the adequacy of both modelling approaches (Figures 2 and 3). Evidence of overdispersion was observed (variance-to-mean ratio = 2.06), supporting the use of count regression models. Multicollinearity was low, with all Variance Inflation Factors (VIFs) below 2. Inspection of Pearson residuals and fitted-value plots indicated acceptable model fit and no major violations of model assumptions.” (Page 13, lines 271-275).*

*See Figures 2 and 3.*
