# Supplementary material for: Determinants of unmet social needs and the role of parental mental health in families from multicultural and regional/rural communities of Australia
Source: PLoS One. 2026 Aug 11;21(8):e0332560. doi: 10.1371/journal.pone.0332560 (PMC13460624; doi:10.1371/journal.pone.0332560)
Supplement: Peer review history

# Author comment: Author response to Decision Letter 0

**DOI:** 10.1371/journal.pone.0332560.r001

1 Sep 2025

Attachment Submitted filename: Response letter.docx

# Peer review: Decision Letter 0

**DOI:** 10.1371/journal.pone.0332560.r002

**Authors:** Werku Etafa

16 Oct 2025

Dear Dr. Eapen,

Thank you for submitting your manuscript to PLOS ONE. After careful consideration, we feel that it has merit but does not fully meet PLOS ONE’s publication criteria as it currently stands. Therefore, we invite you to submit a revised version of the manuscript that addresses the points raised during the review process.

Please submit your revised manuscript by Nov 30 2025 11:59PM. If you will need more time than this to complete your revisions, please reply to this message or contact the journal office at plosone@plos.org. When you're ready to submit your revision, log on to https://www.editorialmanager.com/pone/ and select the 'Submissions Needing Revision' folder to locate your manuscript file.

- A rebuttal letter that responds to each point raised by the academic editor and reviewer(s). You should upload this letter as a separate file labeled 'Response to Reviewers'.
- A marked-up copy of your manuscript that highlights changes made to the original version. You should upload this as a separate file labeled 'Revised Manuscript with Track Changes'.
- An unmarked version of your revised paper without tracked changes. You should upload this as a separate file labeled 'Manuscript'.

If you would like to make changes to your financial disclosure, please include your updated statement in your cover letter. Guidelines for resubmitting your figure files are available below the reviewer comments at the end of this letter.

If applicable, we recommend that you deposit your laboratory protocols in protocols.io to enhance the reproducibility of your results. Protocols.io assigns your protocol its own identifier (DOI) so that it can be cited independently in the future. For instructions see: https://journals.plos.org/plosone/s/submission-guidelines#loc-laboratory-protocols. Additionally, PLOS ONE offers an option for publishing peer-reviewed Lab Protocol articles, which describe protocols hosted on protocols.io. Read more information on sharing protocols at https://plos.org/protocols?utm_medium=editorial-email&utm_source=authorletters&utm_campaign=protocols.

We look forward to receiving your revised manuscript.

Kind regards,

Werku Etafa

Academic Editor

PLOS ONE

Journal Requirements:

When submitting your revision, we need you to address these additional requirements.

1. Please ensure that your manuscript meets PLOS ONE's style requirements, including those for file naming. The PLOS ONE style templates can be found at https://journals.plos.org/plosone/s/file?id=wjVg/PLOSOne_formatting_sample_main_body.pdf and https://journals.plos.org/plosone/s/file?id=ba62/PLOSOne_formatting_sample_title_authors_affiliations.pdf

2. Your ethics statement should only appear in the Methods section of your manuscript. If your ethics statement is written in any section besides the Methods, please move it to the Methods section and delete it from any other section. Please ensure that your ethics statement is included in your manuscript, as the ethics statement entered into the online submission form will not be published alongside your manuscript.

3. We note that you have indicated that there are restrictions to data sharing for this study. For studies involving human research participant data or other sensitive data, we encourage authors to share de-identified or anonymized data. However, when data cannot be publicly shared for ethical reasons, we allow authors to make their data sets available upon request. For information on unacceptable data access restrictions, please see http://journals.plos.org/plosone/s/data-availability#loc-unacceptable-data-access-restrictions.

Before we proceed with your manuscript, please address the following prompts:

a) If there are ethical or legal restrictions on sharing a de-identified data set, please explain them in detail (e.g., data contain potentially identifying or sensitive patient information, data are owned by a third-party organization, etc.) and who has imposed them (e.g., a Research Ethics Committee or Institutional Review Board, etc.). Please also provide contact information for a data access committee, ethics committee, or other institutional body to which data requests may be sent.

b) If there are no restrictions, please upload the minimal anonymized data set necessary to replicate your study findings to a stable, public repository and provide us with the relevant URLs, DOIs, or accession numbers. Please see http://www.bmj.com/content/340/bmj.c181.long for guidelines on how to de-identify and prepare clinical data for publication. For a list of recommended repositories, please see https://journals.plos.org/plosone/s/recommended-repositories. You also have the option of uploading the data as Supporting Information files, but we would recommend depositing data directly to a data repository if possible.

Please update your Data Availability statement in the submission form accordingly.

4. In the online submission form, you indicated that data are available upon reasonable request. Data collected for this study will be shared upon reasonable request to the corresponding author (v.eapen@unsw.edu.au)

All PLOS journals now require all data underlying the findings described in their manuscript to be freely available to other researchers, either 1. In a public repository, 2. Within the manuscript itself, or 3. Uploaded as supplementary information.

This policy applies to all data except where public deposition would breach compliance with the protocol approved by your research ethics board. If your data cannot be made publicly available for ethical or legal reasons (e.g., public availability would compromise patient privacy), please explain your reasons on resubmission and your exemption request will be escalated for approval.

5. We note that the grant information you provided in the ‘Funding Information’ and ‘Financial Disclosure’ sections do not match.

When you resubmit, please ensure that you provide the correct grant numbers for the awards you received for your study in the ‘Funding Information’ section.

6. Thank you for stating the following financial disclosure:

This study was supported through the NSW Health COVID-19 Research Grants Round 2, following an independent peer-review process, and delivered in partnership with the University of New South Wales, South Western Sydney Local Health District, Murrumbidgee Local Health District, NSW Ministry of Health, Sydney Children’s Hospital Randwick, Western Sydney University, Ingham Institute for Applied Medical Research, Black Dog Institute, Uniting, and Karitane. The funding body had no role in the study design, data collection, analysis, interpretation, or in the writing of this manuscript. VE is supported by National Health and Medical Research Council (NHMRC) Investigator Grant #2033610

Please state what role the funders took in the study. If the funders had no role, please state: "The funders had no role in study design, data collection and analysis, decision to publish, or preparation of the manuscript."

If this statement is not correct you must amend it as needed.

Please include this amended Role of Funder statement in your cover letter; we will change the online submission form on your behalf.

7. Please amend either the title on the online submission form (via Edit Submission) or the title in the manuscript so that they are identical.

8. Please amend the manuscript submission data (via Edit Submission) to include author Blaise Di Mento.

9. Please include a caption for figure 1.

10. We note that there is identifying data in the Supporting Information file <Detailed Study protocol.docx>. Due to the inclusion of these potentially identifying data, we have removed this file from your file inventory. Prior to sharing human research participant data, authors should consult with an ethics committee to ensure data are shared in accordance with participant consent and all applicable local laws.

Data sharing should never compromise participant privacy. It is therefore not appropriate to publicly share personally identifiable data on human research participants. The following are examples of data that should not be shared:

-Name, initials, physical address

-Ages more specific than whole numbers

-Internet protocol (IP) address

-Specific dates (birth dates, death dates, examination dates, etc.)

-Contact information such as phone number or email address

-Location data

-ID numbers that seem specific (long numbers, include initials, titled “Hospital ID”) rather than random (small numbers in numerical order)

Data that are not directly identifying may also be inappropriate to share, as in combination they can become identifying. For example, data collected from a small group of participants, vulnerable populations, or private groups should not be shared if they involve indirect identifiers (such as sex, ethnicity, location, etc.) that may risk the identification of study participants.

Additional guidance on preparing raw data for publication can be found in our Data Policy (https://journals.plos.org/plosone/s/data-availability#loc-human-research-participant-data-and-other-sensitive-data) and in the following article: http://www.bmj.com/content/340/bmj.c181.long.

Please remove or anonymize all personal information (<specific identifying information in file to be removed>), ensure that the data shared are in accordance with participant consent, and re-upload a fully anonymized data set. Please note that spreadsheet columns with personal information must be removed and not hidden as all hidden columns will appear in the published file.

11. Please include captions for your Supporting Information files at the end of your manuscript, and update any in-text citations to match accordingly. Please see our Supporting Information guidelines for more information: http://journals.plos.org/plosone/s/supporting-information.

12. If the reviewer comments include a recommendation to cite specific previously published works, please review and evaluate these publications to determine whether they are relevant and should be cited. There is no requirement to cite these works unless the editor has indicated otherwise.

[Note: HTML markup is below. Please do not edit.]

Reviewers' comments:

Reviewer's Responses to Questions

**Comments to the Author**

1. Is the manuscript technically sound, and do the data support the conclusions?

Reviewer #1: Yes

**********

2. Has the statistical analysis been performed appropriately and rigorously?

Reviewer #1: No

**********

3. Have the authors made all data underlying the findings in their manuscript fully available?

The PLOS Data policy

Reviewer #1: Yes

**********

4. Is the manuscript presented in an intelligible fashion and written in standard English?

Reviewer #1: Yes

**********

Reviewer #1: Dear Editor,

I write to submit my review report on the manuscript titled “Determinants of unmet social needs and the role of parental mental health in families from multicultural and regional/rural communities of Australia.”

The study explored the determinants of social care needs and the associated clinical

characteristics such as parental mental health among families from multicultural and

regional/rural communities of Australia

Comments

Methods

1.The authors stated that the presence of unmet needs was dichotomized into two categories: no unmet needs (WE-CARE score = 0) and one or more unmet needs (WE-CARE score ≥ 1). They then fitted a binary logistic regression model to assess the determinants. Sensitivity analyses must be conducted when the outcome is treated as a count (i.e., the number of unmet needs) and modeled using either a Poisson or a negative binomial regression model. You lose so much information if we re-categorize count outcomes. For instance, 2, 3, 4, 5, 6 or higher number of unmet needs are still classified as 1 and logistic regression will not help isolate the differences in the number of unmet needs

2.For a baseline data, the design is basically cross-sectional and the preferred effect size of interest should be prevalence ratio not odds ratio(Barros & Hirakata, 2003). You can generate prevalence ratio for binary outcomes using log-binomial models, modified Poisson (Poisson with robust standard errors), and Cox-proportional models with robust variance estimation(Barros & Hirakata, 2003; Coutinho, Scazufca, & Menezes, 2008; Martinez et al., 2017; Talbot, Mésidor, Chiu, Simard, & Sirois, 2022). Usually, researchers think that you can only model a binary outcome with only probit and logistic regression models and Poisson is for modeling only count outcomes. Poisson and Negative binomial regression models with robust standard errors can be used to model both count and binary outcomes and here is the list of references in the medical literature where different authors compare Poisson with robust standard error, log-binomial models, and logistic regression models, etc. when they analyzed BINARY OUTCOMES using cross-sectional data. Conducting a sensitivity analysis that reports the odds ratio and prevalence ratio from a binary outcome may enhance the paper by treating the outcome measure as a count

References

Bastos, L. S., Oliveira, R. D. V. C. D., & Velasque, L. D. S. (2015). Obtaining adjusted prevalence ratios from logistic regression models in cross-sectional studies. Cadernos de saude publica, 31, 487-495.

Gnardellis, C., Notara, V., Papadakaki, M., Gialamas, V., & Chliaoutakis, J. (2022). Overestimation of relative risk and prevalence ratio: misuse of logistic modeling. Diagnostics, 12(11), 2851

Coutinho, L., Scazufca, M., & Menezes, P. R. (2008). Methods for estimating prevalence ratios in cross-sectional studies. Revista de saude publica, 42, 992-998.

Barros, A. J., & Hirakata, V. N. (2003). Alternatives for logistic regression in cross-sectional studies: an empirical comparison of models that directly estimate the prevalence ratio. BMC medical research methodology, 3(1), 1-13.

Wolkewitz, M., Bruckner, T., & Schumacher, M. (2007). Accurate variance estimation for prevalence ratios. Methods of Information in Medicine, 46(05), 567-571.

3.The authors indicated that univariate linear regression analysis was used to determine the factors independently associated with the risk of unmet needs. Why linear regression when the outcome measure is binary? Is it univariate logistic regression or linear regression, especially when you reported an odds ratio? Clarify

4.The statement “the multivariate binary logistic regression models” should rather be multivariable binary logistic regression model. Multivariate mean modelling two or more dependent variables (outcome measures) simultaneously. Kindly revise

5.Put a footnote of the meaning of n (%) or M(SD) under Table 1

6. What was the overall prevalence of unmet needs and the corresponding 95% confidence interval at baseline and how does it vary across the background characteristics. This important information is missing in the manuscript

**********

what does this mean?). If published, this will include your full peer review and any attached files.

If you choose “no”, your identity will remain anonymous but your review may still be made public.

**Do you want your identity to be public for this peer review?**  For information about this choice, including consent withdrawal, please see our Privacy Policy

Reviewer #1: No

**********

[NOTE: If reviewer comments were submitted as an attachment file, they will be attached to this email and accessible via the submission site. Please log into your account, locate the manuscript record, and check for the action link "View Attachments". If this link does not appear, there are no attachment files.]

While revising your submission, please upload your figure files to the Preflight Analysis and Conversion Engine (PACE) digital diagnostic tool, https://pacev2.apexcovantage.com/. PACE helps ensure that figures meet PLOS requirements. To use PACE, you must first register as a user. Registration is free. Then, login and navigate to the UPLOAD tab, where you will find detailed instructions on how to use the tool. If you encounter any issues or have any questions when using PACE, please email PLOS at figures@plos.org. Please note that Supporting Information files do not need this step.

# Author comment: Author response to Decision Letter 1

**DOI:** 10.1371/journal.pone.0332560.r003

10 Nov 2025

Point-by-point response to Reviewers’ comments

Dear Editor,

We thank you and the reviewers for your time in reviewing our paper and providing valuable comments that have helped to improve the current version. We have carefully considered the comments and responded to all the items. We hope the manuscript after careful revisions meet your high standards. Below we provide the point-by-point responses. All modifications in the manuscript have been highlighted using tracked changes.

Editor’s comments

1. Please ensure that your manuscript meets PLOS ONE's style requirements, including those for file naming. The PLOS ONE style templates can be found at https://journals.plos.org/plosone/s/file?id=wjVg/PLOSOne_formatting_sample_main_body.pdf and https://journals.plos.org/plosone/s/file?id=ba62/PLOSOne_formatting_sample_title_authors_affiliations.pdf

Author response: We have carefully formatted the manuscript as per PLOS ONE's style requirements.

1. Your ethics statement should only appear in the Methods section of your manuscript. If your ethics statement is written in any section besides the Methods, please move it to the Methods section and delete it from any other section. Please ensure that your ethics statement is included in your manuscript, as the ethics statement entered into the online submission form will not be published alongside your manuscript.

Author response: We have now moved the ethics statement to the methods section as requested (See Page 7, Lines 128-134).

2. We note that you have indicated that there are restrictions to data sharing for this study. For studies involving human research participant data or other sensitive data, we encourage authors to share de-identified or anonymized data. However, when data cannot be publicly shared for ethical reasons, we allow authors to make their data sets available upon request. For information on unacceptable data access restrictions, please see http://journals.plos.org/plosone/s/data-availability#loc-unacceptable-data-access-restrictions.

Author response: We have revised the data sharing statement in the manuscript (See Page 18, Lines 371-380).

3. Before we proceed with your manuscript, please address the following prompts:

a) If there are ethical or legal restrictions on sharing a de-identified data set, please explain them in detail (e.g., data contain potentially identifying or sensitive patient information, data are owned by a third-party organization, etc.) and who has imposed them (e.g., a Research Ethics Committee or Institutional Review Board, etc.). Please also provide contact information for a data access committee, ethics committee, or other institutional body to which data requests may be sent.

b) If there are no restrictions, please upload the minimal anonymized data set necessary to replicate your study findings to a stable, public repository and provide us with the relevant URLs, DOIs, or accession numbers. Please see http://www.bmj.com/content/340/bmj.c181.long for guidelines on how to de-identify and prepare clinical data for publication. For a list of recommended repositories, please see https://journals.plos.org/plosone/s/recommended-repositories. You also have the option of uploading the data as Supporting Information files, but we would recommend depositing data directly to a data repository if possible.

Please update your Data Availability statement in the submission form accordingly.

Author response: We have revised the data sharing statement in the manuscript (See Page 18, Lines 371-380).

4. In the online submission form, you indicated that data are available upon reasonable request. Data collected for this study will be shared upon reasonable request to the corresponding author (v.eapen@unsw.edu.au)

All PLOS journals now require all data underlying the findings described in their manuscript to be freely available to other researchers, either 1. In a public repository, 2. Within the manuscript itself, or 3. Uploaded as supplementary information.

This policy applies to all data except where public deposition would breach compliance with the protocol approved by your research ethics board. If your data cannot be made publicly available for ethical or legal reasons (e.g., public availability would compromise patient privacy), please explain your reasons on resubmission and your exemption request will be escalated for approval.

Author response: We have revised the data sharing statement in the manuscript (See Page 18, Lines 371-380).

5. We note that the grant information you provided in the ‘Funding Information’ and ‘Financial Disclosure’ sections do not match.

Author response: We have now updated this information to match in both sections.

6. When you resubmit, please ensure that you provide the correct grant numbers for the awards you received for your study in the ‘Funding Information’ section.

Author response: We have now updated this information for both NSW Health COVID-19 Research Grants Round 2 and National Health and Medical Research Council (NHMRC) Investigator Grant. We have included the application number for the NSW Health COVID-19 Research Grants Round 2 as there was no specific grant identifier number provided (See Pages 18-19, Lines 392-402).

7. Thank you for stating the following financial disclosure:

This study was supported through the NSW Health COVID-19 Research Grants Round 2, following an independent peer-review process, and delivered in partnership with the University of New South Wales, South Western Sydney Local Health District, Murrumbidgee Local Health District, NSW Ministry of Health, Sydney Children’s Hospital Randwick, Western Sydney University, Ingham Institute for Applied Medical Research, Black Dog Institute, Uniting, and Karitane. The funding body had no role in the study design, data collection, analysis, interpretation, or in the writing of this manuscript. VE is supported by National Health and Medical Research Council (NHMRC) Investigator Grant #2033610

Please state what role the funders took in the study. If the funders had no role, please state: "The funders had no role in study design, data collection and analysis, decision to publish, or preparation of the manuscript."

If this statement is not correct you must amend it as needed.

Please include this amended Role of Funder statement in your cover letter; we will change the online submission form on your behalf.

Author response: We have now added the above sentence about the role of funders in the manuscript (See Pages 19, Lines 399-402) and the cover letter.

8. Please amend either the title on the online submission form (via Edit Submission) or the title in the manuscript so that they are identical.

Author response: We have now updated this information to match in both sections.

9. Please amend the manuscript submission data (via Edit Submission) to include author Blaise Di Mento.

Author response: We have now updated this information.

10. Please include a caption for figure 1.

Author response: We have now included a caption for figure 1 (See Page 34).

11. We note that there is identifying data in the Supporting Information file <Detailed Study protocol.docx>. Due to the inclusion of these potentially identifying data, we have removed this file from your file inventory. Prior to sharing human research participant data, authors should consult with an ethics committee to ensure data are shared in accordance with participant consent and all applicable local laws.

Data sharing should never compromise participant privacy. It is therefore not appropriate to publicly share personally identifiable data on human research participants. The following are examples of data that should not be shared:

-Name, initials, physical address

-Ages more specific than whole numbers

-Internet protocol (IP) address

-Specific dates (birth dates, death dates, examination dates, etc.)

-Contact information such as phone number or email address

-Location data

-ID numbers that seem specific (long numbers, include initials, titled “Hospital ID”) rather than random (small numbers in numerical order)

Data that are not directly identifying may also be inappropriate to share, as in combination they can become identifying. For example, data collected from a small group of participants, vulnerable populations, or private groups should not be shared if they involve indirect identifiers (such as sex, ethnicity, location, etc.) that may risk the identification of study participants.

Additional guidance on preparing raw data for publication can be found in our Data Policy (https://journals.plos.org/plosone/s/data-availability#loc-human-research-participant-data-and-other-sensitive-data) and in the following article: http://www.bmj.com/content/340/bmj.c181.long.

Please remove or anonymize all personal information (<specific identifying information in file to be removed>), ensure that the data shared are in accordance with participant consent, and re-upload a fully anonymized data set. Please note that spreadsheet columns with personal information must be removed and not hidden as all hidden columns will appear in the published file.

Author response: We thank the Editorial team for this feedback. We confirm that the detailed study protocol document only contained names of the investigatory team and not research participants’ data. We have now removed the details of the investigatory team as well.

12. Please include captions for your Supporting Information files at the end of your manuscript, and update any in-text citations to match accordingly. Please see our Supporting Information guidelines for more information: http://journals.plos.org/plosone/s/supporting-information.

Author response: We have now added the captions for the supporting information files in text and at the end of the manuscript (See Page 34).

13. If the reviewer comments include a recommendation to cite specific previously published works, please review and evaluate these publications to determine whether they are relevant and should be cited. There is no requirement to cite these works unless the editor has indicated otherwise.

Author response: We thank the Editorial team for this information.

Reviewers' comments:

Reviewer #1:

Dear Editor,

I write to submit my review report on the manuscript titled “Determinants of unmet social needs and the role of parental mental health in families from multicultural and regional/rural communities of Australia.” The study explored the determinants of social care needs and the associated clinical characteristics such as parental mental health among families from multicultural and regional/rural communities of Australia

Comments

Methods

1.The authors stated that the presence of unmet needs was dichotomized into two categories: no unmet needs (WE-CARE score = 0) and one or more unmet needs (WE-CARE score ≥ 1). They then fitted a binary logistic regression model to assess the determinants. Sensitivity analyses must be conducted when the outcome is treated as a count (i.e., the number of unmet needs) and modeled using either a Poisson or a negative binomial regression model. You lose so much information if we re-categorize count outcomes. For instance, 2, 3, 4, 5, 6 or higher number of unmet needs are still classified as 1 and logistic regression will not help isolate the differences in the number of unmet needs

Author response: Thank you for your feedback. To conform with reporting standards for cross-sectional studies, we have revised the statistical methods. The data was overdispersed, hence univariable and multivariable analyses were conducted with negative binomial regression. We check for overdispersion using the ‘overdisp’ module in Stata (See Methods section – Page 4, Lines 63-66 and Pages 9-10, Lines 183-196).

Modified sections: “Univariable negative binomial regression analysis was used to determine the factors independently associated with increased rate of unmet needs, where unmet needs were treated as count data for each unmet need. Factors included sociodemographic, sociocultural, and clinical indicators where unmet social needs at baseline (model 1), 6 months (model 2), and 12 months (model 3) were the respective outcomes. The analyses repeated the above three models in a multivariable negative binomial regression analysis with all the above exposures at once for each of the models. Negative binomial regression was used due to overdispersion seen between the outcome and each of the above factors. All analyses were undertaken in Stata v19 (StataCorp. 2025. Stata Statistical Software: Release 19. College Station, TX: StataCorp LLC.)”

Please also see revised analysis in Tables 2 and 3 (Pages 27-34), results section (Pages 11-12, Lines 215-257) and the discussion section (Page 14, 286-293).

2.For a baseline data, the design is basically cross-sectional and the preferred effect size of interest should be prevalence ratio not odds ratio(Barros & Hirakata, 2003). You can generate prevalence ratio for binary outcomes using log-binomial models, modified Poisson (Poisson with robust standard errors), and Cox-proportional models with robust variance estimation(Barros & Hirakata, 2003; Coutinho, Scazufca, & Menezes, 2008; Martinez et al., 2017; Talbot, Mésidor, Chiu, Simard, & Sirois, 2022). Usually, researchers think that you can only model a binary outcome with only probit and logistic regression models and Poisson is for modeling only count outcomes. Poisson and Negative binomial regression models with robust standard errors can be used to model both count and binary outcomes and here is the list of references in the medical literature where different authors compare Poisson with robust standard error, log-binomial models, and logistic regression models, etc. when they analyzed BINARY OUTCOMES using cross-sectional data. Conducting a sensitivity analysis that reports the odds ratio and prevalence ratio from a binary outcome may enhance the paper by treating the outcome measure as a count

References

Bastos, L. S., Oliveira, R. D. V. C. D., & Velasque, L. D. S. (2015). Obtaining adjusted prevalence ratios from logistic regression models in cross-sectional studies. Cadernos de saude publica, 31, 487-495.

Gnardellis, C., Notara, V., Papadakaki, M., Gialamas, V., & Chliaoutakis, J. (2022). Overestimation of relative risk and prevalence ratio: misuse of logistic modeling. Diagnostics, 12(11), 2851

Coutinho, L., Scazufca, M., & Menezes, P. R. (2008). Methods for estimating prevalence ratios in cross-sectional studies. Revista de saude publica, 42, 992-998.

Barros, A. J., & Hirakata, V. N. (2003). Alternatives for logistic regression in cross-sectional studies: an empirical comparison of models that directly estimate the prevalence ratio. BMC medical research methodology, 3(1), 1-13.

Wolkewitz, M., Bruckner, T., & Schumacher, M. (2007). Accurate variance estimation for prevalence ratios. Methods of Information in Medicine, 46(05), 567-571.

Author response: Thank you for your feedback. To conform with reporting standards for cross-sectional studies, we have revised the statistical methods. The data was overdispersed, hence univariable and multivariable analyses were conducted with negative binomial regression. We check for overdispersion using the ‘overdisp’ module in Stata (See Methods section – Page 4, Lines 63-66 and Pages 9-10, Lines 183-196).

Modified sections: “Univariable negative binomial regression analysis was used to determine the factors independently associated with increased rate of unmet needs, where unmet needs were treated as count data for each unmet need. Factors included sociodemographic, sociocultural, and clinical indicators where unmet social needs at baseline (model 1), 6 months (model 2), and 12 months (model 3) were the respective outcomes. The analyses repeated the above three models in a multivariable negative binomial regression analysis with all the above exposures at once for each of the models. Negative binomial regression was used due to overdispersion seen between the outcome and each of the above factors. All analyses were undertaken in Stata v19 (StataCorp. 2025. Stata Statistical Software: Release 19. College Station, TX: StataCorp

Attachment Submitted filename: Response to Reviewers.docx

# Peer review: Decision Letter 1

**DOI:** 10.1371/journal.pone.0332560.r004

**Authors:** Werku Etafa

12 Dec 2025

Dear Dr.  Eapen,

Thank you for submitting your manuscript to PLOS ONE. After careful consideration, we feel that it has merit but does not fully meet PLOS ONE’s publication criteria as it currently stands. Therefore, we invite you to submit a revised version of the manuscript that addresses the points raised during the review process.

Please submit your revised manuscript by Jan 26 2026 11:59PM. If you will need more time than this to complete your revisions, please reply to this message or contact the journal office at plosone@plos.org. When you're ready to submit your revision, log on to https://www.editorialmanager.com/pone/ and select the 'Submissions Needing Revision' folder to locate your manuscript file.

- A letter that responds to each point raised by the academic editor and reviewer(s). You should upload this letter as a separate file labeled 'Response to Reviewers'.
- A marked-up copy of your manuscript that highlights changes made to the original version. You should upload this as a separate file labeled 'Revised Manuscript with Track Changes'.
- An unmarked version of your revised paper without tracked changes. You should upload this as a separate file labeled 'Manuscript'.

If applicable, we recommend that you deposit your laboratory protocols in protocols.io to enhance the reproducibility of your results. Protocols.io assigns your protocol its own identifier (DOI) so that it can be cited independently in the future. For instructions see: https://journals.plos.org/plosone/s/submission-guidelines#loc-laboratory-protocols. Additionally, PLOS ONE offers an option for publishing peer-reviewed Lab Protocol articles, which describe protocols hosted on protocols.io. Read more information on sharing protocols at https://plos.org/protocols?utm_medium=editorial-email&utm_source=authorletters&utm_campaign=protocols.

We look forward to receiving your revised manuscript.

Kind regards,

Werku Etafa

Academic Editor

PLOS One

Journal Requirements:

1. If the reviewer comments include a recommendation to cite specific previously published works, please review and evaluate these publications to determine whether they are relevant and should be cited. There is no requirement to cite these works unless the editor has indicated otherwise.

2. Please review your reference list to ensure that it is complete and correct. If you have cited papers that have been retracted, please include the rationale for doing so in the manuscript text, or remove these references and replace them with relevant current references. Any changes to the reference list should be mentioned in the rebuttal letter that accompanies your revised manuscript. If you need to cite a retracted article, indicate the article’s retracted status in the References list and also include a citation and full reference for the retraction notice.

[Note: HTML markup is below. Please do not edit.]

Reviewer's Responses to Questions

**Comments to the Author**

Reviewer #1: All comments have been addressed

**********

2. Is the manuscript technically sound, and do the data support the conclusions?

Reviewer #1: Yes

**********

3. Has the statistical analysis been performed appropriately and rigorously?

Reviewer #1: Yes

**********

4. Have the authors made all data underlying the findings in their manuscript fully available?

The PLOS Data policy

Reviewer #1: Yes

**********

5. Is the manuscript presented in an intelligible fashion and written in standard English?

Reviewer #1: Yes

**********

Reviewer #1: Almost all my comments have been addressed adequately except this one. "Author response: Thank you for your feedback. We have now provided row percentages for one or more unmet needs and no unmet needs for each demographic characteristic. We believe that writing 95% confidence intervals in a descriptive table of summary statistics is inappropriate use of statistical inference due to multiple testing concerns (See Pages 24-26)". Unfortunately that is not the case as this has nothing to do with testing the difference. Is purely estimation. No p-values. Point estimates alone does not fully tell us what happens in the population. Kindly estimate the CIs

**********

what does this mean?). If published, this will include your full peer review and any attached files.

If you choose “no”, your identity will remain anonymous but your review may still be made public.

**Do you want your identity to be public for this peer review?**  For information about this choice, including consent withdrawal, please see our Privacy Policy

Reviewer #1: No

**********

[NOTE: If reviewer comments were submitted as an attachment file, they will be attached to this email and accessible via the submission site. Please log into your account, locate the manuscript record, and check for the action link "View Attachments". If this link does not appear, there are no attachment files.]

To ensure your figures meet our technical requirements, please review our figure guidelines: https://journals.plos.org/plosone/s/figures

You may also use PLOS’s free figure tool, NAAS, to help you prepare publication quality figures: https://journals.plos.org/plosone/s/figures#loc-tools-for-figure-preparation.

NAAS will assess whether your figures meet our technical requirements by comparing each figure against our figure specifications.

# Author comment: Author response to Decision Letter 2

**DOI:** 10.1371/journal.pone.0332560.r005

15 Dec 2025

Dear Editor,

We thank you and the reviewers for your time in reviewing our paper and providing valuable comments that have helped to improve the current version. We have carefully considered the comments and responded to all the items. We hope the manuscript after careful revisions meet your high standards. Below we provide the point-by-point responses. All modifications in the manuscript have been highlighted using tracked changes.

Reviewers' comments:

Reviewer #1:

1. Almost all my comments have been addressed adequately except this one. "Author response: Thank you for your feedback. We have now provided row percentages for one or more unmet needs and no unmet needs for each demographic characteristic. We believe that writing 95% confidence intervals in a descriptive table of summary statistics is inappropriate use of statistical inference due to multiple testing concerns (See Pages 24-26)". Unfortunately that is not the case as this has nothing to do with testing the difference. Is purely estimation. No p-values. Point estimates alone does not fully tell us what happens in the population. Kindly estimate the CIs.

Author response: We thank the reviewer for the feedback. We agree that 95% confidence intervals provide information on the precision of descriptive estimates and are not related to hypothesis testing. Accordingly, we have now estimated and reported 95% confidence intervals for the row percentages in the descriptive table (Table 1).

Modifications made: See Table 1.

Attachment Submitted filename: Response_to_Reviewers_auresp_2.docx

# Peer review: Decision Letter 2

**DOI:** 10.1371/journal.pone.0332560.r006

**Authors:** Werku Etafa

24 May 2026

Dear Dr. Eapen,

Thank you for submitting your manuscript to PLOS ONE. After careful consideration, we feel that it has merit but does not fully meet PLOS ONE’s publication criteria as it currently stands. Therefore, we invite you to submit a revised version of the manuscript that addresses the points raised during the review process.

Please submit your revised manuscript by Jul 08 2026 11:59PM. If you will need more time than this to complete your revisions, please reply to this message or contact the journal office at plosone@plos.org. When you're ready to submit your revision, log on to https://www.editorialmanager.com/pone/ and select the 'Submissions Needing Revision' folder to locate your manuscript file.

- A letter that responds to each point raised by the academic editor and reviewer(s). You should upload this letter as a separate file labeled 'Response to Reviewers'.
- A marked-up copy of your manuscript that highlights changes made to the original version. You should upload this as a separate file labeled 'Revised Manuscript with Track Changes'.
- An unmarked version of your revised paper without tracked changes. You should upload this as a separate file labeled 'Manuscript'.

If you would like to make changes to your financial disclosure, please include your updated statement in your cover letter. Guidelines for resubmitting your figure files are available below the reviewer comments at the end of this letter.

If applicable, we recommend that you deposit your laboratory protocols in protocols.io to enhance the reproducibility of your results. Protocols.io assigns your protocol its own identifier (DOI) so that it can be cited independently in the future. For instructions see: https://journals.plos.org/plosone/s/submission-guidelines#loc-laboratory-protocols. Additionally, PLOS ONE offers an option for publishing peer-reviewed Lab Protocol articles, which describe protocols hosted on protocols.io. Read more information on sharing protocols at https://plos.org/protocols?utm_medium=editorial-email&utm_source=authorletters&utm_campaign=protocols.

As the corresponding author, your ORCID iD is verified in the submission system and will appear in the published article. PLOS supports the use of ORCID, and we encourage all coauthors to register for an ORCID iD and use it as well. Please encourage your coauthors to verify their ORCID iD within the submission system before final acceptance, as unverified ORCID iDs will not appear in the published article. *Only*  the individual author can complete the verification step; PLOS staff *cannot*  verify ORCID iDs on behalf of authors.

We look forward to receiving your revised manuscript.

Kind regards,

Werku Etafa

Academic Editor

PLOS One

**Journal Requirements:**

1. If the reviewer comments include a recommendation to cite specific previously published works, please review and evaluate these publications to determine whether they are relevant and should be cited. There is no requirement to cite these works unless the editor has indicated otherwise.

2. Please review your reference list to ensure that it is complete and correct. If you have cited papers that have been retracted, please include the rationale for doing so in the manuscript text, or remove these references and replace them with relevant current references. Any changes to the reference list should be mentioned in the rebuttal letter that accompanies your revised manuscript. If you need to cite a retracted article, indicate the article’s retracted status in the References list and also include a citation and full reference for the retraction notice.

[Note: HTML markup is below. Please do not edit.]

Reviewers' comments:

Reviewer's Responses to Questions

**Comments to the Author**

Reviewer #1: All comments have been addressed

Reviewer #2: (No Response)

**********

2. Is the manuscript technically sound, and do the data support the conclusions?

Reviewer #1: Yes

Reviewer #2: Yes

**********

3. Has the statistical analysis been performed appropriately and rigorously?

Reviewer #1: Yes

Reviewer #2: Yes

**********

4. Have the authors made all data underlying the findings in their manuscript fully available?

The PLOS Data policy

Reviewer #1: Yes

Reviewer #2: Yes

**********

5. Is the manuscript presented in an intelligible fashion and written in standard English?

Reviewer #1: Yes

Reviewer #2: Yes

**********

Reviewer #1: Delete the third column of Table 1. I was referring to row percentages and corresponding 95% confidence interval for the outcome measure for each of the background characteristics. You have now provided that in column 5 and 7, perfect. Just delete the third column of Table 1. Ideally you should have one table for the background characteristics and another Table for the prevalence (row percentages) and confidence interval estimates for each background characteristics but you lumped them in Table 1 with the pure descriptive statistics. That was why I said in my previous review to estimate confidence interval for the outcome measure for each background characteristics. Once you delete the third column of Table 1, everything will fall in place. Note that prevalence (row percentages) is referring to the outcome measure. For instance, among those who experience premature birth, what percentage had say one or more

unmet needs, the answer is 61.0% [95%CI: 54.9-66.5]. This is what I was referring to. You must categorize the continuous variables like age into meaningful and relevant policy-based age categories and estimate the prevalence of experiencing one or more unmet needs across each category. This applies to other continuous variables in Table 1. Another way to do this is to keep Table 1 but only column 1 and 2 which is purely descriptive and delete other remaining columns. Then you create table 2 and titled that Table " Prevalence of one or more unmet needs by background characteristics". So for Table 2, you will have two columns, Column 1 is the background characteristics and column 2 is Prevalence [95% CI]. This is very informative and will improve the manuscript

Reviewer #2: The statistical section is generally well structured and appropriate for a public health study, but there are several strengths and areas that could be improved for clarity, rigor, and reporting quality. The study clearly defines the primary outcome as: Unmet social care needs, Measured using the WE CARE survey and Treated as a count variable. This is statistically appropriate because unmet needs are naturally count data.

The author’s state: As the data were over dispersed, univariable and multivariable negative binomial regression models were applied…. This is a strong methodological choice for count data.

The study used Univariable analysis and Multivariable negative binomial regression which helps Identify crude associations and Adjust for confounding variables. And good practice in epidemiological and clinical studies.

Statistical Areas for Improvement

a)Randomized Controlled Trial Design Not Fully Reflected in Analysis

The study is described as secondary analysis of a randomized controlled trial, however, the statistical section does not explain: Whether treatment group allocation was adjusted for, whether intervention effects were controlled and whether clustering/repeated measures were considered. This is important because repeated observations over time may violate independence assumptions.

If possible better approach or if the authors could consider: Mixed-effects negative binomial regression, Generalized Estimating Equations (GEE) since measurements were taken repeatedly over time.

b)Missing Model Diagnostics:

If the article does discuss Goodness-of-fit, Residual analysis, multicollinearity and influential observations. And I will recommended diagnostic contains Variance Inflation Factor (VIF), Residual plots, AIC/BIC and Pearson residuals

**********

what does this mean?). If published, this will include your full peer review and any attached files.

If you choose “no”, your identity will remain anonymous but your review may still be made public.

**Do you want your identity to be public for this peer review?**  For information about this choice, including consent withdrawal, please see our Privacy Policy

Reviewer #1: No

Reviewer #2: **Yes:**  Shibiru Jabessa

**********

[NOTE: If reviewer comments were submitted as an attachment file, they will be attached to this email and accessible via the submission site. Please log into your account, locate the manuscript record, and check for the action link "View Attachments". If this link does not appear, there are no attachment files.]

To ensure your figures meet our technical requirements, please review our figure guidelines: https://journals.plos.org/plosone/s/figures

You may also use PLOS’s free figure tool, NAAS, to help you prepare publication quality figures: https://journals.plos.org/plosone/s/figures#loc-tools-for-figure-preparation.

NAAS will assess whether your figures meet our technical requirements by comparing each figure against our figure specifications.

# Author comment: Author response to Decision Letter 3

**DOI:** 10.1371/journal.pone.0332560.r007

10 Jun 2026

Point-by-point response to Reviewers’ comments

Dear Editor,

We thank you and the reviewers for your time in reviewing our paper and providing valuable comments that have helped to improve the current version. We have carefully considered the comments and responded to all the items. We hope the manuscript after careful revisions meet your high standards. Below we provide the point-by-point responses. All modifications in the manuscript have been highlighted using tracked changes.

Reviewers' comments:

Reviewer #1

1. Delete the third column of Table 1. I was referring to row percentages and corresponding 95% confidence interval for the outcome measure for each of the background characteristics. You have now provided that in column 5 and 7, perfect. Just delete the third column of Table 1. Ideally you should have one table for the background characteristics and another Table for the prevalence (row percentages) and confidence interval estimates for each background characteristics but you lumped them in Table 1 with the pure descriptive statistics. That was why I said in my previous review to estimate confidence interval for the outcome measure for each background characteristics. Once you delete the third column of Table 1, everything will fall in place. Note that prevalence (row percentages) is referring to the outcome measure. For instance, among those who experience premature birth, what percentage had say one or more unmet needs, the answer is 61.0% [95%CI: 54.9-66.5]. This is what I was referring to. You must categorize the continuous variables like age into meaningful and relevant policy-based age categories and estimate the prevalence of experiencing one or more unmet needs across each category. This applies to other continuous variables in Table 1. Another way to do this is to keep Table 1 but only column 1 and 2 which is purely descriptive and delete other remaining columns. Then you create table 2 and titled that Table " Prevalence of one or more unmet needs by background characteristics". So for Table 2, you will have two columns, Column 1 is the background characteristics and column 2 is Prevalence [95% CI]. This is very informative and will improve the manuscript

Author response: We thank the reviewer for this feedback. We have revised the tables accordingly. Specifically, we deleted the third column from Table 1 and retained Table 1 as a descriptive summary of the sample characteristics. As suggested, we have also created a new Table 2 titled “Prevalence of One or More Unmet Needs by Background Characteristics,” which presents the row prevalence estimates with corresponding 95% confidence intervals across each background characteristic category. In addition, to provide a more complete representation of the outcome distribution, Table 2 now includes both participants with one or more unmet needs and those with no unmet needs.

We have also categorised continuous variables into meaningful policy-relevant categories to improve interpretability and alignment as suggested.

Modifications made: See Tables 1 and 2.

Reviewer #2

The statistical section is generally well structured and appropriate for a public health study, but there are several strengths and areas that could be improved for clarity, rigor, and reporting quality. The study clearly defines the primary outcome as: Unmet social care needs, Measured using the WE CARE survey and Treated as a count variable. This is statistically appropriate because unmet needs are naturally count data. The author’s state: As the data were over dispersed, univariable and multivariable negative binomial regression models were applied…. This is a strong methodological choice for count data. The study used Univariable analysis and Multivariable negative binomial regression which helps Identify crude associations and adjust for confounding variables. And good practice in epidemiological and clinical studies.

Statistical Areas for Improvement

a) Randomized Controlled Trial Design Not Fully Reflected in Analysis. The study is described as secondary analysis of a randomized controlled trial, however, the statistical section does not explain: Whether treatment group allocation was adjusted for, whether intervention effects were controlled and whether clustering/repeated measures were considered. This is important because repeated observations over time may violate independence assumptions. If possible better approach or if the authors could consider: Mixed-effects negative binomial regression, Generalized Estimating Equations (GEE) since measurements were taken repeatedly over time.

Author response: We thank the reviewer for this feedback. We agree that the longitudinal nature of the data requires consideration of within-subject correlation arising from repeated measurements over time. To address this, we conducted a Generalized Estimating Equations (GEE) negative binomial regression analysis in addition to the multivariable negative binomial regression model. The GEE approach accounts for the non-independence of repeated observations and provides population-averaged estimates while accommodating the overdispersed count nature of the outcome. We have also removed the univariable regression tables and results to avoid too many tables.

Modifications made:

Methods section – “To assess the robustness of these findings and to account for repeated measurements over time, a Generalised Estimating Equations (GEE) negative binomial model with an exchangeable correlation structure was additionally fitted. This model incorporated all three time points (baseline, 6 months, and 12 months) and accounted for within‑participant clustering. Treatment group allocation (intervention vs control) was included as a covariate to reflect the underlying randomised controlled trial design.” (Page 10, lines 196-201).

Results section – “To assess the robustness of these findings and account for repeated measurements over time, a GEE negative binomial model was fitted (Table 4). The GEE model incorporated all three time points simultaneously and adjusted for within participant clustering. The results were highly consistent with the standalone negative binomial models. Significant predictors in the GEE model included parental psychological distress, number of child developmental concerns, CALD background, lower parental education, marital status, and parental age. The intervention group showed a significantly lower rate of unmet needs (AIRR 0.75, 95% CI: 0.60, –0.95), consistent with the direction of effects observed in the original models.” (Page 13, lines 260-267).

Discussion section – “Additionally, findings of the GEE model further suggest that these factors may have a sustained influence on families’ unmet social needs over time, rather than reflecting challenges at baseline.” (Page 14, lines 287-289).

See Table 4 (Page 36).

b) Missing Model Diagnostics: If the article does discuss Goodness-of-fit, Residual analysis, multicollinearity and influential observations. And I will recommended diagnostic contains Variance Inflation Factor (VIF), Residual plots, AIC/BIC and Pearson residuals

Author response: We have now added model diagnostics such as variance-to-mean ratio, Variance Inflation Factors (VIFs), Pearson residuals and fitted-value plots.

Modifications made:

Methods section – “Model diagnostics were conducted to assess the suitability of the count regression models. Overdispersion was evaluated using the variance-to-mean ratio, multicollinearity was assessed using Variance Inflation Factors (VIFs), and model fit was examined through inspection of Pearson residuals and fitted-value plots.” (Page 10, lines 203-206).

Results section – “Diagnostic evaluation supported the adequacy of both modelling approaches (Figures 2 and 3). Evidence of overdispersion was observed (variance-to-mean ratio = 2.06), supporting the use of count regression models. Multicollinearity was low, with all Variance Inflation Factors (VIFs) below 2. Inspection of Pearson residuals and fitted-value plots indicated acceptable model fit and no major violations of model assumptions.” (Page 13, lines 271-275).

See Figures 2 and 3.

Attachment Submitted filename: Response_to_Reviewers_auresp_3.docx

# Peer review: Decision Letter 3

**DOI:** 10.1371/journal.pone.0332560.r008

**Authors:** Werku Etafa

26 Jul 2026

Determinants of unmet social needs and the role of parental mental health in families from multicultural and regional/rural communities of Australia

PONE-D-25-45300R3

Dear Dr. Eapen,

We’re pleased to inform you that your manuscript has been judged scientifically suitable for publication and will be formally accepted for publication once it meets all outstanding technical requirements.

Within one week, you’ll receive an e-mail detailing the required amendments. When these have been addressed, you’ll receive a formal acceptance letter and your manuscript will be scheduled for publication.

An invoice will be generated when your article is formally accepted. Please note, if your institution has a publishing partnership with PLOS and your article meets the relevant criteria, all or part of your publication costs will be covered. Please make sure your user information is up-to-date by logging into Editorial Manager at Editorial Manager® and clicking the ‘Update My Information' link at the top of the page. For questions related to billing, please contact billing support.

If your institution or institutions have a press office, please notify them about your upcoming paper to help maximize its impact. If they’ll be preparing press materials, please inform our press team as soon as possible -- no later than 48 hours after receiving the formal acceptance. Your manuscript will remain under strict press embargo until 2 pm Eastern Time on the date of publication. For more information, please contact onepress@plos.org.

Kind regards,

Werku Etafa

Academic Editor

PLOS One

Additional Editor Comments (optional):

Reviewers' comments:

Reviewer's Responses to Questions

**Comments to the Author**

Reviewer #1: All comments have been addressed

**********

2. Is the manuscript technically sound, and do the data support the conclusions?

Reviewer #1: Yes

**********

3. Has the statistical analysis been performed appropriately and rigorously?

Reviewer #1: Yes

**********

4. Have the authors made all data underlying the findings in their manuscript fully available?

The PLOS Data policy

Reviewer #1: Yes

**********

5. Is the manuscript presented in an intelligible fashion and written in standard English?

Reviewer #1: Yes

**********

Reviewer #1: The authors have comprehensively addressed all the comments and concerns raised in my previous review of the manuscript

**********

what does this mean?). If published, this will include your full peer review and any attached files.

If you choose “no”, your identity will remain anonymous but your review may still be made public.

**Do you want your identity to be public for this peer review?**  For information about this choice, including consent withdrawal, please see our Privacy Policy

Reviewer #1: No

**********

# Editor report: Acceptance letter

**DOI:** 10.1371/journal.pone.0332560.r009

**Authors:** Werku Etafa

PONE-D-25-45300R3

PLOS One

Dear Dr. Eapen,

I'm pleased to inform you that your manuscript has been deemed suitable for publication in PLOS One. Congratulations! Your manuscript is now being handed over to our production team.

At this stage, our production department will prepare your paper for publication. This includes ensuring the following:

* All references, tables, and figures are properly cited

* All relevant supporting information is included in the manuscript submission,

* There are no issues that prevent the paper from being properly typeset

You will receive further instructions from the production team, including instructions on how to review your proof when it is ready. Please keep in mind that we are working through a large volume of accepted articles, so please give us a few days to review your paper and let you know the next and final steps.

Lastly, if your institution or institutions have a press office, please let them know about your upcoming paper now to help maximize its impact. If they'll be preparing press materials, please inform our press team within the next 48 hours. Your manuscript will remain under strict press embargo until 2 pm Eastern Time on the date of publication. For more information, please contact onepress@plos.org.

You will receive an invoice from PLOS for your publication fee after your manuscript has reached the completed accept phase. If you receive an email requesting payment before acceptance or for any other service, this may be a phishing scheme. Learn how to identify phishing emails and protect your accounts at https://explore.plos.org/phishing.

If we can help with anything else, please email us at customercare@plos.org.

Thank you for submitting your work to PLOS ONE and supporting open access.

Kind regards,

PLOS ONE Editorial Office Staff

on behalf of

Mr. Werku Etafa

Academic Editor

PLOS One
